# Feature weighted models to address lineage dependency in drug-resistance prediction from *Mycobacterium tuberculosis* genome sequences

**DOI:** 10.1093/bioinformatics/btad428

**Published:** 2023-07-10

**Authors:** Nina Billows, Jody E Phelan, Dong Xia, Yonghong Peng, Taane G Clark, Yu-Mei Chang

**Affiliations:** Department of Comparative Biomedical Sciences, Royal Veterinary College, London, United Kingdom; Alan Turing Institute, British Library, London, United Kingdom; Department of Infectious and Tropical Diseases, London School of Hygiene & Tropical Medicine, London, United Kingdom; Department of Comparative Biomedical Sciences, Royal Veterinary College, London, United Kingdom; Department of Computing and Mathematics, Manchester Metropolitan University, Manchester, United Kingdom; Department of Infectious and Tropical Diseases, London School of Hygiene & Tropical Medicine, London, United Kingdom; Department of Epidemiology and Population Health, London School of Hygiene & Tropical Medicine, London, United Kingdom; Department of Comparative Biomedical Sciences, Royal Veterinary College, London, United Kingdom

## Abstract

**Motivation:**

Tuberculosis (TB) is caused by members of the *Mycobacterium tuberculosis* complex (MTBC), which has a strain- or lineage-based clonal population structure. The evolution of drug-resistance in the MTBC poses a threat to successful treatment and eradication of TB. Machine learning approaches are being increasingly adopted to predict drug-resistance and characterize underlying mutations from whole genome sequences. However, such approaches may not generalize well in clinical practice due to confounding from the population structure of the MTBC.

**Results:**

To investigate how population structure affects machine learning prediction, we compared three different approaches to reduce lineage dependency in random forest (RF) models, including stratification, feature selection, and feature weighted models. All RF models achieved moderate-high performance (area under the ROC curve range: 0.60–0.98). First-line drugs had higher performance than second-line drugs, but it varied depending on the lineages in the training dataset. Lineage-specific models generally had higher sensitivity than global models which may be underpinned by strain-specific drug-resistance mutations or sampling effects. The application of feature weights and feature selection approaches reduced lineage dependency in the model and had comparable performance to unweighted RF models.

**Availability and implementation:**

https://github.com/NinaMercedes/RF_lineages.

## 1 Introduction

Tuberculosis (TB), caused by *Mycobacterium tuberculosis*, has a significant impact on public health worldwide, resulting in 1.6 million deaths in 2021 alone (World Health Organization, 2022). The primary treatment of TB is to use a combination of first-line drugs including rifampicin (RIF), isoniazid (INH), ethambutol (EMB), and pyrazinamide (PZA). However, multi-drug resistant TB (MDR-TB) (resistance to RIF and INH) has developed and second-line therapies are increasingly required for effective treatment of TB (World Health Organization, 2022). Previously, second-line treatments included fluoroquinolones [ofloxacin (OFL), moxifloxacin (MOX), levofloxacin (LEV)], second-line injectables [amikacin (AMI), capreomycin (CAP), kanamycin (KAN), streptomycin (STM)], and other drugs [cycloserine (CYS), ethionamide (ETD), para-aminosalicylic acid (PAS)] (WHO 2022). More recently, WHO updated the treatment guidelines due to the need for shorter and effective treatments for drug-susceptible and MDR-TB (WHO 2021). Recent changes to treatment guidelines and drug-resistant phenotype classification emphasizes the ongoing development of the drug-resistant TB problem. Therefore, it is important to gain insight into the biological drivers of resistance with a view to improve TB treatment and diagnosis.

Machine learning (ML) algorithms offer a new method to address the drug-resistant TB problem by simultaneously predicting drug-resistant phenotypes and exploring the genomic variation that underpins drug-resistance (Niehaus *et al.* 2014, Yang *et al.* 2018, Kouchaki *et al.* 2019). Numerous traditional ML approaches have been applied to predict drug-resistance such as logistic regression, decision trees, random forests (RFs), and gradient boosted trees (Niehaus *et al.* 2014, Yang *et al.* 2018, Deelder *et al.* 2019, Kouchaki *et al.* 2019, Libiseller-Egger *et al.* 2020, Kouchaki *et al.* 2020). Although such models have achieved moderate-high performance, their application in clinical settings is hindered due to lack of interpretability.

It is suggested that the ideal solution would be for ML algorithms to predict resistance using only mutations that are causative of drug-resistance. This would facilitate the surveillance of drug-resistance mutations. However, most published models rely on non-causative mutations to boost the predictive performance of the model (Deelder *et al.* 2019). For example, co-occurrent resistant mutations that cause resistance to a different drug are often assigned high importance and contribute to improved performance for some drugs (Deelder *et al.* 2019). Likewise, it is hypothesized that ML prediction is confounded by population structure, contributing to high importance of lineage-specific mutations across models (Deelder *et al.* 2019, Yang *et al.* 2019, Libiseller-Egger *et al.* 2020). Even so, the mechanisms that underly drug-resistance are complex and can differ between lineages, indicating that some lineage-specific mutations may play a role in drug-resistance (Wu *et al.* 2013, Oppong *et al.* 2019). Therefore, it is important to determine how population structure affects model performance and interpretability.

The *M.tuberculosis* complex (MTBC) is a group of genetically related *Mycobacterium* species that are responsible for causing TB. The MTBC has a highly clonal population structure with no ongoing horizontal gene transfer and low recombination rate (Hershberg *et al.* 2008, Gagneux 2018, Ngabonziza *et al.* 2020). It is comprised of several human and animal adapted lineages, including *M.tuberculosis* sensu stricto (Lineages 1–4 and 7), *Mycobacterium var. africanum* (Lineages 5–6), and at least nine zoonotic lineages (Coll *et al.* 2014, Napier *et al.* 2020). Additionally, new Lineages 8 and 9 have recently been described (Napier *et al.* 2020, Coscolla *et al.* 2021). Whilst Lineages 2 (East Asian) and 4 (Euro-American) are more widespread, the remaining lineages are geographically isolated, suggesting strains have co-evolved with human populations (Hershberg *et al.* 2008, Gagneux 2012, 2018). Population structure is of particular importance in the context of resistance prediction for several reasons. Firstly, most ML models assume that samples are independent which may be invalid due to the ancestral relationships between isolates. This can lead to spurious genotype–phenotype associations because of confounding. In addition, it has previously been shown that the performance of resistance prediction can vary across countries and lineages (Mahé *et al.* 2019, World Health, Organization 2018). This variation may be due to the genetic background of MTBC lineages which can vary in transmission, virulence, and drug-resistance (Niemann *et al.* 2010, Krishnan *et al.* 2011, Karmakar *et al.* 2019, Oppong *et al.* 2019). Poor performance can also stem from a sampling effect whereby more prevalent lineages make up most existing datasets, leading to a lack of knowledge of drug-resistance mutations in under-sampled lineages and poor generalization of predictive models. Consequently, it is important to explore how lineage dependency affects the prediction of resistant phenotypes across the MTBC.

Confounding from population structure has yet to be fully addressed in ML prediction. Previous studies have applied a weight to each sample according to its clade size and strain prevalence (Lees *et al.* 2020, Nguyen *et al.* 2020). However, in some circumstances, this led to reduced model performance and the effectiveness was dependent on the complexity of the population structure. In contrast, population structure has been adjusted for in genome wide association studies (GWAS) using a variety of methods, including the use of kinship matrices in linear mixed models, covariates derived from principal component analysis (PCA), multidimensional scaling on pairwise distances, and de Bruijn graphs (Zhou and Stephens 2012, Earle *et al.* 2016, Phelan *et al.* 2016, Coll *et al.* 2018, Jaillard *et al.* 2018, Lees *et al.* 2018, Oppong *et al.* 2019). Furthermore, convergence analysis tests have been developed to identify homoplastic mutations enriched in resistant branches across a phylogenetic tree (Farhat *et al.* 2013, Phelan *et al.* 2016, Collins and Didelot 2018). Such analyses inherently account for confounding from clonal population structure and have been essential for improving our understanding of the mechanisms that underpin drug-resistance in the MTBC. In contrast, there is no standard approach to account for population structure in ML models. It is important to address this limitation for ML models to generalize and perform optimally across MTBC lineages.

Given the existing limitations previously reported for ‘off-the-shelf’ ML algorithms, we explore the effects of reducing lineage dependency using RF models. The RF model is a non-parametric tree ensemble algorithm that combines the output of multiple decision trees to make a prediction (Breiman 2001). Notably, RF models are favoured as they are interpretable and can capture feature interactions (Nembrini *et al.* 2018). The strong phylogeographical associations exhibited by the MTBC may indicate the need for models that predict resistance for each lineage separately. Therefore, we first measured how RF models perform using stratified datasets that are comprised of the most prevalent lineages of the MTBC (Lineages 2 and 4) in comparison to a global version based on all lineages. Alternatively, ML methods that take advantage of evolutionary convergence would intrinsically account for population structure and prioritize mutations that have evolved independently multiple times. Consequently, we devised a method to weight features according to their homoplasy distribution to indicate the probability that it will be used as a split-variable in the model. We hypothesized that the feature weighted approach can improve the robustness of resistance prediction without jeopardizing the performance of the model. This insight has important implications for genotype–phenotype predictions carried out across a wide range of disciplines that are frequently confounded by population structure, including infectious disease and genomic medicine.

## 2 Materials and methods

### 2.1 Whole genome sequencing data

A dataset that was curated prior to this study was used for the analysis (Coll *et al.* 2015, Phelan *et al.* 2019). The dataset is comprised of whole genome sequences (WGS) and drug susceptibility test (DST) data for 18 396 MTBC isolates and was collated from previously published studies. WGS was performed using Illumina sequencing and were processed using methods that have previously been described (Coll *et al.* 2015, Phelan *et al.* 2019). In brief, raw reads were aligned to the H37Rv reference genome (Genbank accession NC_000962.3) using BWA mem algorithm and variants (single-nucleotide polymorphisms [SNPs]; insertions/deletions (indels)] were called using SAMtools/BCFtools and GATK software (McKenna *et al.* 2010, Li 2011). Missing genotypes were assigned if the total depth of coverage was 20 or at least 75% of the total coverage was not reported for by one nucleotide. Samples or variant sites were removed if greater than 10% of genotypes were assigned as missing. Missing genotypes were infrequent and assumed to be missing at random. Missing genotypes were assigned using a phylogenetic-based imputation method. Allele frequency was calculated using VCFtools (v1.9) (Danecek *et al.* 2011).

### 2.2 DST data

Binary DST data were obtained using WHO recommended protocols from clinical isolates that were retrieved from individual patients. For our analysis, susceptibility to 13 drugs including first-line drugs (INH, RIF, EMB, PZA), fluoroquinolones (OFL, MOX), aminoglycosides (AMI, CAP, KAN, STM), and other drugs (CYS, ETD, PAS) was considered. In addition, MDR was also predicted for comparison and was defined as resistance to both INH and RIF. DST data for each individual drug were not available for all isolates and there were varying degrees of completeness across all drugs. Therefore, samples with missing phenotypes for each drug were removed per analysis.

### 2.3 Training and testing datasets

The global TB dataset (*n* = 18 396) was split into training and testing datasets which were used to train and test the performance of the RF model, respectively. As several lineages are represented in the dataset, some of which are known to contain lineage-specific drug-resistance mutations, a stratified sampling approach was taken to ensure the training and testing datasets for the global model contained equal proportions of resistant and susceptible isolates derived from each lineage across all 14 phenotypes. The majority (80%) of the data was used to train the algorithm and the remaining subset (20%) was used for testing purposes. We also included combined and separate training and testing datasets for Lineages 2 and 4 to assess how RF models perform over individual lineages.

### 2.4 Random forest training and predictive performance

All RF models were implemented using the Ranger package in R and used to predict binary DST phenotypes from genome variants (Wright and Ziegler 2017). Methods to account for lineage dependency were compared (Supplementary Table SA). Model hyperparameters, such as split rule were optimized using 5-fold cross-validation using the grid search approach available in the caret package in R (Kuhn 2008). Default settings were used for *mtry* (square root of number of features), and *minimum node size* (1) as preliminary analysis had shown that they were optimal for classification. Additionally, we used 1000 trees (*num.trees*) and a maximum depth of 10 (*max.depth*) consistent with previous analyses (Libiseller-Egger *et al.* 2020). To address imbalances in the number of susceptible and resistant isolates, resistant and susceptible phenotypes were weighted inversely proportional to their respective frequencies (weights summed to one).

Three different strategies to account for lineage-specific variants were used: (i) stratified analysis applied to global, combined (Lineages 2 and 4) and lineage-specific (Lineage 2 or 4) data; (ii) feature selection model: excluding lineage-specific variants (score < 2); and (iii) feature weighted model. The *split.select.weights* option implemented by Ranger software was used to weight features in the model, as demonstrated by a previous study (Oskooei *et al.* 2019). This provides a probability that the feature will be used for splitting in the RF model. The overall predictive performance was assessed using area under the ROC curve (AUC-ROC), Sensitivity, Specificity and F1 score. The framework used to generate these results is summarized in Supplementary SB.

### 2.5 Population structure and feature weight calculation

Two methods were used to infer the population structure of the global dataset. Firstly, phylogenetic trees were obtained from a genome-wide SNP alignment using FastTree (v2.1 double precision) software with a Generalized Time Reversible (GTR) substitution model. Branch lengths were rescaled to compute a Gamma20-based likelihood (Price *et al.* 2010). SNPs in hypervariable regions, including PE/PPE genes, were excluded from the alignment. Phylogenetic trees for training and testing datasets were built independently and rooted on an *Mycobacterium canetti* isolate. Trees were pre-processed using the Ape package in R (v3.6.1) (Paradis and Schliep 2019). After pre-processing the phylogenetic tree of the training dataset, ancestral states were reconstructed using maximum likelihood and parsimony methods in the Phangorn package (Schliep 2011). Results between parsimony and maximum likelihood methods were comparable and all following results were obtained using the parsimony approach. The site-wise parsimony score for each variant was estimated by Fitch’s parsimony algorithm using the Phangorn package in R (Schliep 2011). Parsimony scores were defined as the minimum number of state changes that are required to explain the genotypes observed at the tips of the tree. In the feature weighted models, normalized parsimony scores were used to weight features in the RF model. Additionally, population structure of sub-lineages was also inferred by PCA using PCAtools (v3.15).

### 2.6 Feature selection

Variants (SNPs and indels) in 29 candidate genes encoded in a binary format were used as features in the analysis. Candidate genes were selected in line with the TB-Profiler database, a mutation catalogue that has undergone expert curation (Supplementary SC) (Phelan *et al.* 2019). This includes variants that are listed in the WHO drug-resistance mutation catalogue for TB (Walker *et al.* 2022). For the purpose of comparison, an additional feature selection method was used whereby features with a parsimony score of <2 were removed from the dataset. Such features would otherwise have a weight of 0 in the weighted model. Removed features were also compared to existing MTBC barcodes that contain lineage-specific variants to ensure that all lineage defining mutations were removed (Coll *et al.* 2014, Napier *et al.* 2020, Freschi *et al.* 2021). This removal was to assess the performance, interpretability, and robustness of a feature weighted model in comparison to this traditional feature selection method [unweighted RF (parsimony score <2)] where lineage-specific variants are removed.

### 2.7 Ranking feature importance and feature interactions

Feature importance was assessed using Gini importance due to its superiority in capturing interactions between features when compared to permutation importance (Nembrini *et al.* 2018). To establish a threshold for the ‘most important’ features in the model, the analysis was rerun, and features were recurrently eliminated until the maximum AUC-ROC was reached. Variants were converted to HGVS format using SNPEff software (v4.3) and compared to *M.tuberculosis* H37Rv genome to infer variant functional class and effects (Danecek *et al.* 2011). All features were compared to the TB-profiler database and literature, as well as a list of lineage-specific variants to classify them as either known drug-resistance mutation or co-occurring mutation (causes resistance to another drug) (Phelan *et al.* 2019). Variants were also assigned as ‘lineage’ according to their phylogenetic distribution in the training dataset. This was to account for lineage effects that might be observed where a variant is highly prevalent in one lineage but observed rarely in other lineages which may be indicative of confounding. Variants were considered as putative novel drug-resistance if they were included in the ‘most important’ features in the model, >90% samples that contain the variant were resistant, and in a known drug-resistance gene.

Feature interactions were also explored. The occurrence of parent–child node interactions was summed up across 1000 trees in the RF. The most frequent interactions (top 1%) were identified using frequency graphs. Interactions were classified in a similar manner to features, as described above. For example, if the parent node was a known drug-resistance variant and the child node was lineage-specific, the interaction would be labelled as ‘Known: Lineage’. Interactions between drug-resistance mutations and compensatory mutations were also examined.

## 3 Results

### 3.1 Genomic and phenotypic data

**Figure 1.**
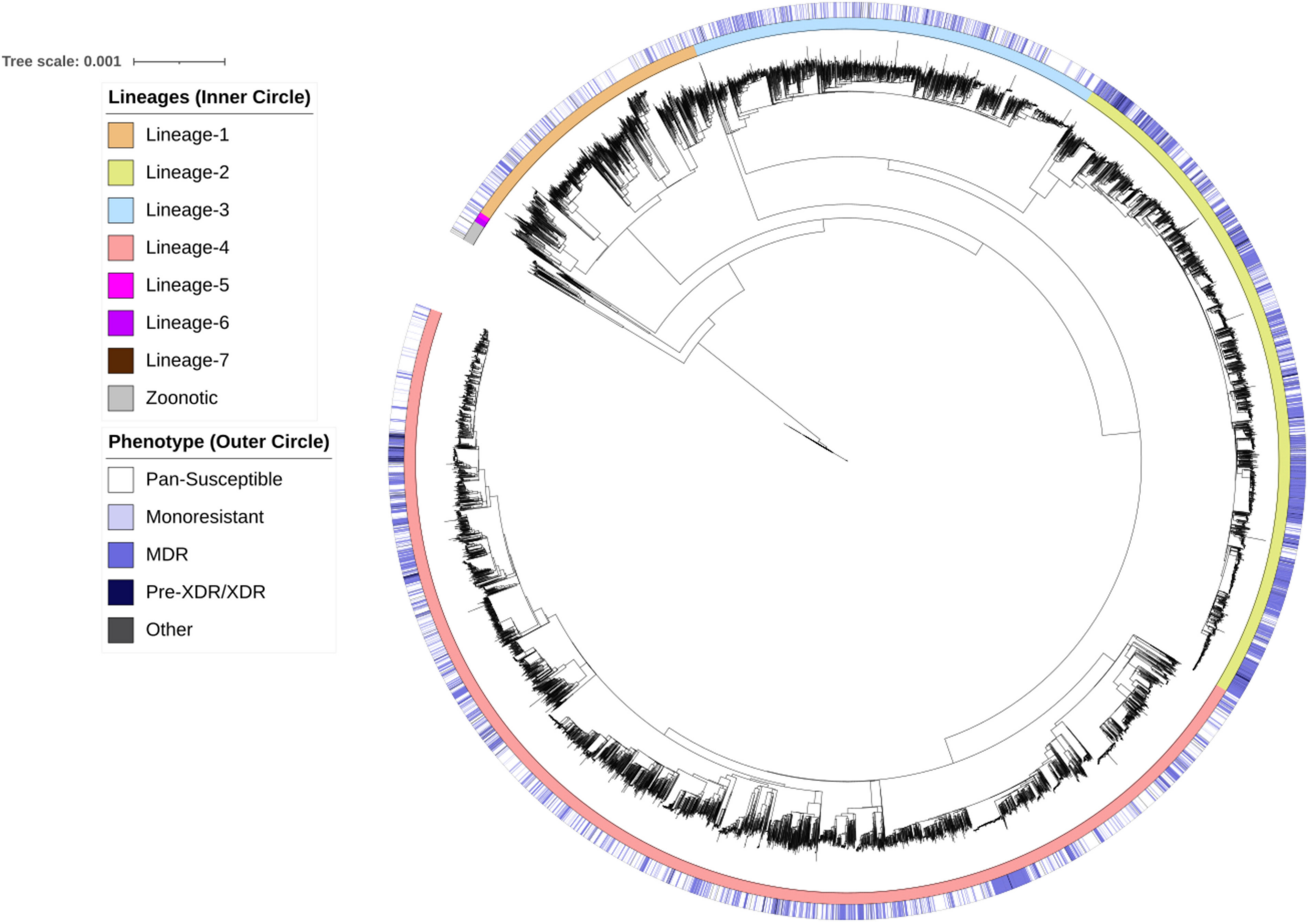
Phylogenetic analysis of the training dataset annotated with corresponding lineage and drug-resistant phenotype. The training dataset was comprised of 14 724 MTBC isolates that belong to Lineages 1–7 and zoonotic species (inner ring). The outer ring shows the composite drug-resistant phenotypes which are shaded according to increasing severity of resistance, including pan-susceptible, mono-resistant, MDR, pre-XDR, and other.

WGS were available for 18 396 *M.tuberculosis* isolates. Most isolates belong to Lineages 2 (*N* = 4605, 25.0%) and 4 (*N* = 8875, 48.2%), whilst fewer isolates represented the remaining lineages. Most isolates were pan-susceptible (*N* = 10 976, 59.7%), but a considerable proportion were RIF-resistant (*N* = 5403, 29.9%) and MDR (*N* = 4608, 25.1%). Phenotypic data were most complete for first-line drugs RIF (*N* = 18 087, 98%), INH (*N* = 17 895, 97.0%), EMB (*N* = 16576, 90.0%), and PZA (*N* = 13248, 72.0%). However, data were limited for most second-line drugs, especially for PAS and CYS (<10%). Phylogenetic analysis of the training dataset revealed isolates cluster according to lineage (Fig. 1). Resistant phenotypes were unevenly distributed throughout lineages (Supplementary SD). A larger percentage of Lineage 2 (60.3%) isolates were MDR in comparison to Lineage 4 (22.32%) (Supplementary SD). PCA also revealed isolates cluster according to lineage and sub-lineage and greater diversity was observed for Lineage 4 (Supplementary SE).

### 3.2 Data predictive performance of global, combined, and lineage-specific models

**Figure 2.**
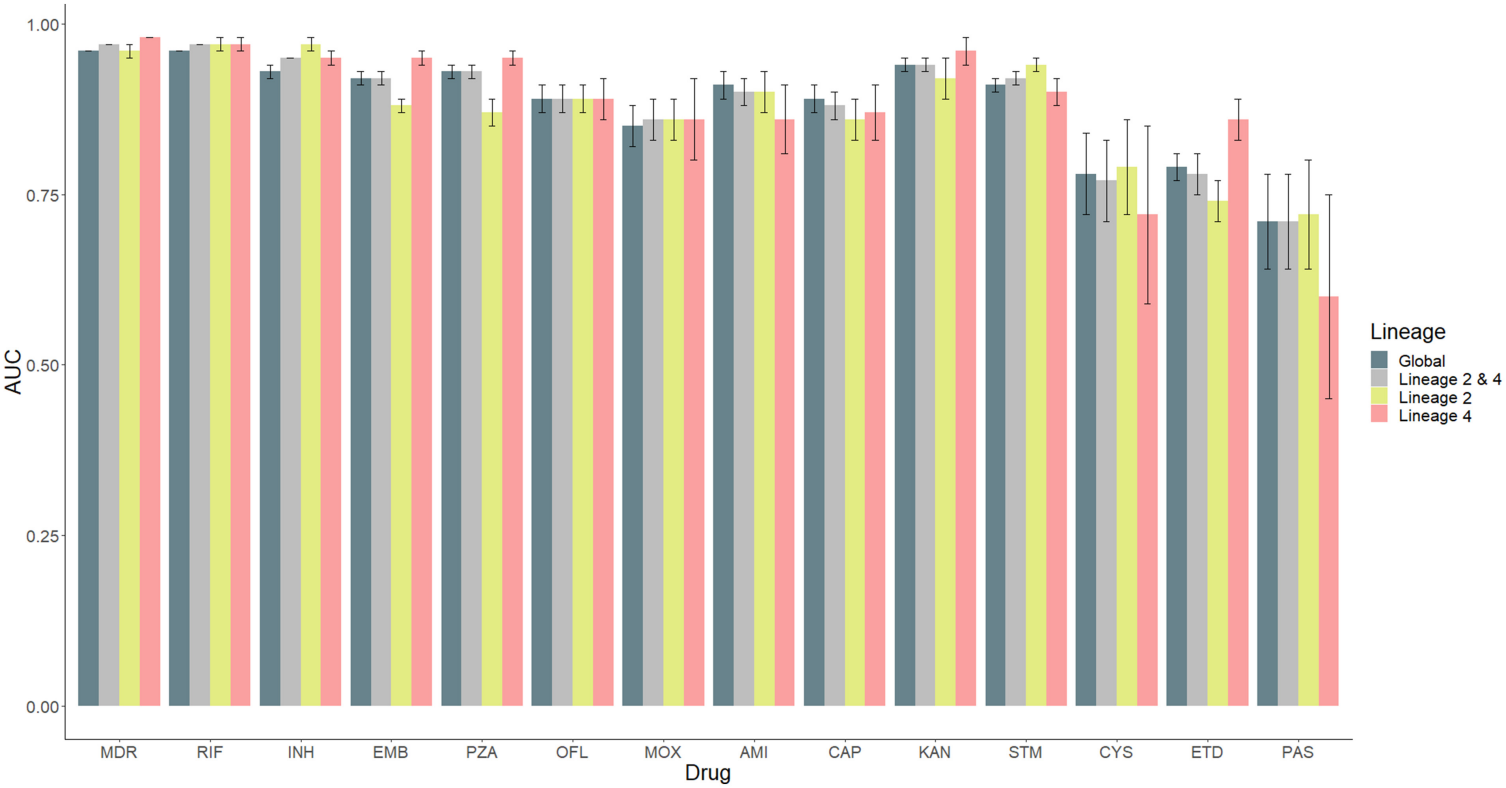
AUC-ROC of lineage-specific, combined, and global RF models predicting 14 drug-resistant phenotypes. AUC-ROC for each drug-resistant phenotype is shown. Bars are filled according to lineages included in the analysis. Error bars show 95% confidence intervals for each prediction.

We first assessed the effectiveness of stratification for dealing with lineage dependency by comparing the AUC-ROC, sensitivity, and specificity of the RF models. Overall, the predictive performance of the RF model varied across the global and lineage-specific models for each drug (Supplementary SD). As observed in previous studies, the AUC-ROC was generally higher for first-line (>0.85) than second-line drugs. AUC-ROC was especially limited for drugs with fewer samples, including ETD [Global AUC-ROC = 0.79 (0.77–0.81)], CYS [Global AUC-ROC = 0.78 (0.72–0.84)], and PAS [Global AUC-ROC = 0.71 (0.64–0.78)]. The optimal performance of RF models differed between drugs. The AUC-ROC for global and combined datasets (Lineages 2 and 4) were comparable (Fig. 2). This was unsurprising given that the global dataset is mostly comprised of isolates from Lineages 2 and 4. For AMI [Global AUC-ROC = 0.91 (0.89–0.93)] and CAP [Global AUC-ROC = 0.89 (0.87–0.91)], the global model displayed the highest AUC-ROC (Fig. 2). In contrast, there was higher AUC-ROC for Lineage 2 (INH, STM, CYS and PAS) and Lineage 4 (MDR, EMB, PZA, KAN, and ETD) (Fig. 2). Global and lineage-specific RF models performed similarly for RIF, OFL, and MOX (Supplementary SD). These results indicated that performance across drugs can vary depending on the strain diversity within the training and testing datasets.

This observation was further exemplified by the variation in sensitivity observed. Lineage-specific models tended to outperform global models in terms of sensitivity (Supplementary SD). Highest sensitivity was observed in the Lineage 2 specific model for MDR [0.89 (0.87–0.90)], RIF [0.88 (0.87–0.89)], INH [0.85 (0.84–0.87)], MOX [0.78 (0.72–0.83)], STM [0.87 (0.85–0.89)], and PAS [0.40 (0.28–0.54)]. The higher sensitivity may be driven in part by the larger number of resistant samples in Lineage 2 available for these drugs (Supplementary SD). A larger sample size may include a higher number of known drug-resistance mutations that can drive improvements in sensitivity. Meanwhile, highest sensitivity was reported for Lineage 4 in PZA [0.87 (0.84–0.89)], KAN [0.84 (0.79–0.88)], CYS [0.52 (0.32–0.72)], and ETD [0.70 (0.63–0.75)]. Specificity was generally high across all models, with a slight trade-off with sensitivity (Supplementary SD). Collectively, this indicates that the performance of RF model prediction is highly dependent on the lineages and drug-resistant phenotypes represented in the dataset.

### 3.3 Identification of known and putative lineage-specific resistant mutations

To identify what was driving the variation in performance, we measured the feature importance across lineage-specific, combined, and global models (Supplementary SF). Features were classified as a known drug-resistance mechanism if they had previously been incorporated in the TB-Profiler database (Phelan *et al.* 2019). In addition, mutations were also labelled as having ‘co-occurring’ or ‘lineage’ effects. The feature importance threshold differed between models meaning that the optimal performance was achieved using a varying number of mutations (Supplementary SF). Despite undergoing stratification, high importance was still assigned to variants with lineage effects (Supplementary SF). This highlights that confounding from population structure is a deep-rooted issue and that confounding occurs at the sub-lineage level. This was especially noticeable for drugs with limited phenotype data including CYS, ETD, and PAS (Supplementary SF). The number of known drug-resistance mutations identified by lineage-specific and global models also varied. Lineage-specific models were able to capture drug-resistance mutations that are restricted to single lineages. For example, RF models trained on Lineage 2 isolates assigned high importance to known drug-resistance mutations in *ethA*, including Ala381Pro and 1010_1010del that are found exclusively in Lineages 2.2.2 and 2.2.1, respectively (Supplementary SG). We also report variants with lineage-specific associations with drug-resistance that have yet to be described for EMB (*embA* Ala576Thr, Lineage 4.2.2.1) and ETD (*ethR* 579G>C, Lineage 4.3.4.2) (Supplementary SG). However, their role in drug-resistance cannot be fully established based on the outcome of ML models.

### 3.4 Performance of unweighted and feature weighted models

**Figure 3.**
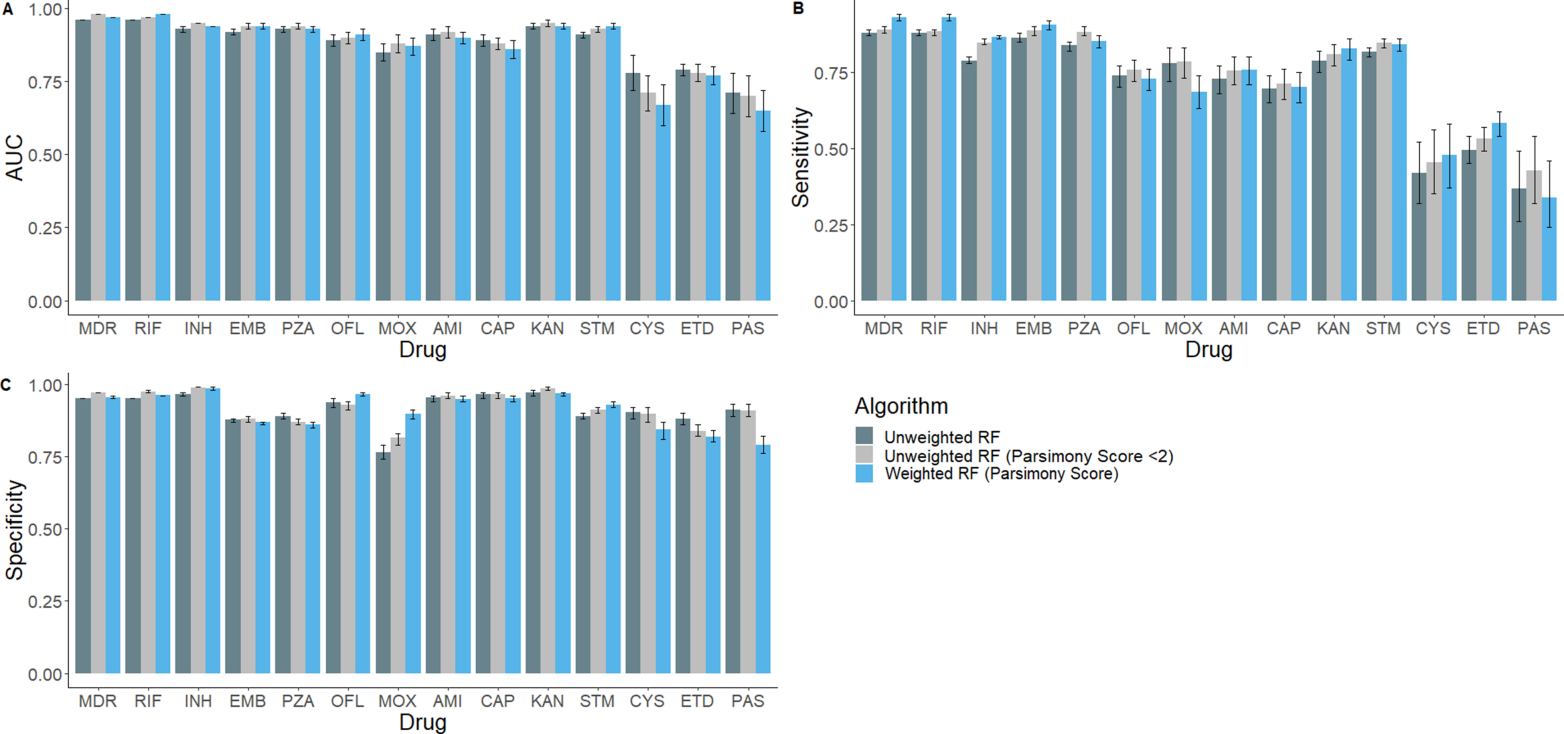
Performance of unweighted and feature weighted RF models predicting 14 drug-resistant phenotypes. AUC-ROC (A), Sensitivity (B), and Specificity (C) for each drug-resistant phenotype is shown. Bars are filled according to feature weight and feature selection method used. Error bars show 95% confidence intervals for prediction. Plots produced using ggplot2 package in R.

Moreover, the feature selection and feature weighted approaches had better or equivalent AUC in comparison to unweighted RF models across all first-line drugs (Supplementary SH). Using either the feature selection approach or feature weighting led to increased or similar sensitivity across all drugs (Fig. 3B). For half of the resistant phenotypes, higher sensitivity was achieved using parsimony score to weight features in the model, including MDR [0.93 (0.92–0.94)], RIF [0.93 (0.92–0.94)], INH [0.87 (0.86–0.87)], EMB [0.91 (0.89–0.92)], KAN [0.83 (0.79–0.86)] (Fig. 3, Supplementary SH). In contrast, the feature selection approach had higher sensitivity for OFL [0.76 (0.72–0.79)], MOX [0.78 (0.73–0.83)], CAP [0.71 (0.66–0.76)], and ETD [0.43 (0.32–0.54)]. This indicated that reducing lineage dependency may not necessarily lead to weaker performance of the global model. Additionally, the performance differed between the choice of approach used to account for lineage. The feature selection approach needs to utilize information from existing knowledge about lineage-specific variants and may miss variants that are not yet defined. The feature weighting approach utilizes all available information from the data and accounts for unknown lineage-specific variants and biases in the distribution of mutations across the phylogeny (Fig. 3). Regardless of the approach used, there was low sensitivity for CYS, ETD and PAS (Supplementary SH).

### 3.5 Feature importance and interactions of feature weighted models

**Figure 4.**
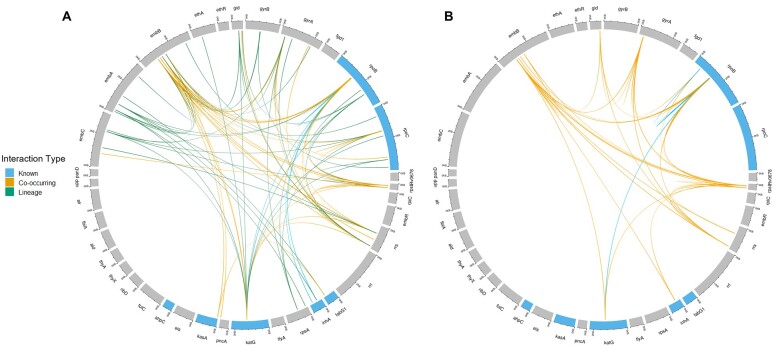
Most frequent interactions (top 1%) observed across 1000 trees in RF model for MDR TB prediction. (A) Most frequent variant–variant interactions in the unweighted RF model. (B) Most frequent variant–variant interactions in the weighted model. Genes known to contain MDR mutations are highlighted. Interactions are classified as a known drug-resistance interaction, co-occurring interaction, and lineage interaction. Interactions were visualized using shinyCircos (Yu et al., 2018).

We assessed the impact of reducing lineage dependency on the model further by evaluating the feature importance and most frequent interactions in the model. The importance of variants that contribute to lineage dependency in the model was mostly reduced using the feature weighted model (Supplementary SI). Across most drugs, the feature selection approach also reduced the importance of clade-specific variants but was less effective when compared to the feature weighted model (Supplementary SI). In some cases, this was advantageous as drug-resistance mutations belonging to a single lineage were ranked highly. This list included a frameshift mutation in *tlyA* 751_752insTG (Lineage 4.3.4.2) (Supplementary SG). The robustness of drug-resistance prediction was analysed by comparing the most frequent interactions in the Unweighted model and Feature Weighted model. The feature weighting method effectively removed all frequent lineage interactions across all drugs (Fig. 4, Supplementary SJ). Within the top 1% of interactions, the number of interactions between known drug-resistance mutations increased using the feature weighted model for the MDR phenotype (Fig. 4). Whilst the feature weighted RF (FW-RF) approach increased the interactions between known drug-resistance mutations, both models were unable to capture interactions between all drug-resistance mutations. Reducing lineage dependency also led to increased reliance on co-occurring mutations associated with resistance to RIF, EMB, PZA, ETD, PAS, and CYS (Supplementary SJ). This is shown further by a higher frequency of co-occurring interactions in the feature weighted model. Additionally, no lineage-specific drug-resistance mutations or putative novel drug-resistance mutations were reported by the feature weighted model.

## 4 Discussion

Confounding from population structure occurs as a result of the highly clonal nature of the MTBC phylogeny which has been driven by asexual reproduction, an absence of horizontal gene transfer and low levels of recombination (Gagneux 2018). Whilst the effects of confounding from population structure are widely considered for GWAS, it remains a key limitation for genotype–phenotype prediction in ML studies. In this study, we addressed bias in ML prediction of *M.tuberculosis* drug-resistant phenotypes that occurs due to population structure. We developed a novel method, Feature Weighted Random Forest (FW-RF), to account for lineage dependency in ML prediction and compare it to ad-hoc approaches, namely stratification and feature selection.

Stratification of the global dataset into lineage-specific (Lineage 2 and Lineage 4 separately) and combined (Lineages 2 and 4 combined) datasets led to varying performance depending on lineages and drug-resistant phenotypes represented in the datasets. This result suggests that resistance to specific antitubercular agents can vary between lineages. Intrinsic differences between MTBC sub-lineages have been explored. The most notable example being the increased transmission of the modern Beijing sub-lineage associated with MDR-TB (Cox *et al.* 2005, Niemann *et al.* 2010, Li *et al.* 2016, Karmakar *et al.* 2019).

This insight has been supported further by evidence of lineage-specific genotypic associations with drug-resistance in separate and combined analysis for major lineages (Oppong *et al.* 2019). Previous analyses have also shown predictions based on profiling tools and molecular diagnostic tests can differ between lineages and countries respectively (World Health, Organization 2018, Mahé *et al.* 2019). This difference may stem from an inconsistent diagnostic and treatment regimen implemented across countries, whereby second-line treatments and new drugs (bedaquiline and delamanid) are excluded from essential medicine lists required for basic healthcare (World Health Organization, 2022). Furthermore, our study showed that lineage-specific models tended to outperform the global and combined models across the drug panel. This result confirms concerns made by previous studies that global models may not necessarily perform and generalize well in clinical practice (Mahé *et al.* 2019). Therefore, future studies should provide an evaluation of performance for individual lineages to indicate the general applicability of ML models. Whilst stratification contributed to improved performance, it does not prevent confounding due to the ancestral relationships between samples occurring at the sub-lineage level. Stratification was also not possible across all lineages because of a limited number of samples being available for the remaining lineages, implying that other approaches are required to handle population structure.

When comparing our novel FW-RF approach to traditional feature selection method, we observed that the removal of lineage-specific mutations, that were defined using predetermined SNP barcodes, may not account for all lineage dependency in the model and also led to the removal of drug-resistance mutations found within specific lineages (Coll *et al.* 2014, Napier *et al.* 2020, Freschi *et al.* 2021). Previously developed barcodes primarily include SNPs and do not include insertions and deletions, larger structural variants, or SNPs in known drug-resistance genes (Coll *et al.* 2014). Therefore, such variants would have been maintained within the set of features. The feature selection process also could be considered too strict due to the complete removal of phylogenetic related features.

The FW-RF model uses feature weights to determine the probability that features will be used as a split variable. The advantage of this approach is that the number of times mutations have evolved independently is taken into account. This observation is more consistent with convergent evolution and selection of drug-resistant mutations. Although the importance of strain-specific drug-resistance mutations will be lowered using this approach, the effect size helps to compensate for its suppression. This enables us to maintain predictive performance whilst improving the interpretability of the model. Whilst this study has primarily focused on feature weighting in the context of RF prediction, feature weights could be applied to several other algorithms, including support vector machines, K-nearest neighbour, neural networks, and learning classifier systems (Urbanowicz and Moore 2015, Chen and Hao 2017, Huang *et al.* 2021). Future research should be carried out to explore feature weighting mechanisms and expert knowledge discovery in prediction tasks further.

Our study supports outcomes from prior research that have shown that confounding variables such as lineage and co-occurring phenotypes boost model performance (Deelder *et al.* 2019, Green *et al.* 2022). We find that despite reducing lineage dependency using the FW-RF model, the performance was maintained across most drugs. Whilst this could be due to greater importance of known drug-resistance mutations, it may also be caused by confounding from co-occurring resistance to other drugs which could indicate overfitting to the dataset (Deelder *et al.* 2022). This emphasizes the importance of addressing confounding in ML prediction, even though such features may increase performance, to make interpretable and robust predictions.

Additionally, we found that variation in performance across the stratified datasets was underpinned by the representation of drug-resistant phenotypes across sub-lineages, as well as drug-resistance mutations, that have emerged in a single lineage. For example, *ethA* Ala381Pro and 1010_1010del were identified as important features for predicting ETD resistance in Lineage 2. ETD is a pro-drug activated by the mycobacterial monooxygenase EthA. Mutations in *ethA* prevent the activation of ETD, some of which have been reported as lineage-specific (Coll *et al.* 2018, Alame Emane *et al.* 2021). We also report putative novel mutations in *ethR* (*579G>C*), a transcriptional regulator of *ethA*, as well as *embA* (Ala576Thr), which encodes the drug target of EMB, in Lineage 4. Lineage 4 is considered to have greater strain diversity than Lineage 2 which has facilitated the discovery of novel drug-resistance mechanisms (Oppong *et al.* 2019). Despite this, previous epidemiological and *in vitro* studies have suggested that Lineage 2 isolates are at a greater risk of developing drug-resistance than Lineage 4 (Torres Ortiz *et al.* 2021). Consequently, it is thought isolates belonging to Lineage 2 are predisposed to developing resistance due to their genomic background (Torres Ortiz *et al.* 2021). Compensatory mutations, such as those in *rpoC*, were also reported as high-ranking features across all models regardless of lineages represented in the dataset. Compensatory mutations are considered to alleviate fitness costs associated with drug-resistance and some studies suggest association with transmission (Casali *et al.* 2014). There is conflicting evidence as to whether compensatory mutations vary between strain types and their role in drug-resistance is not fully understood (de Vos *et al.* 2013, Casali *et al.* 2014, Li *et al.* 2016, Liu *et al.* 2018, Merker *et al.* 2018). This questions whether concentrating solely on causal variants overlooks the role that other mutations play in drug-resistance, including compensatory and lineage-specific mutations. For example, lineage-specific mutations may influence the transmission of MDR strains. In such cases, lineage-specific mutations can directly impact drug-resistance or have broader implications on bacterial fitness (Shah *et al.* 2017). As a whole, this indicates that the molecular mechanisms that underpin drug-resistance are complex and are dependent on an interplay between genetic background, epistasis, and fitness (Borrell and Gagneux 2011).

We note that there are several limitations and areas of further research that would enhance the outcome of this study. Firstly, we only consider two major lineages of the MTBC in lineage-specific models. We also note the limited number of samples for second-line drugs within these lineages. A larger number of samples from under-sampled lineages would facilitate research into improving current knowledge surrounding the evolution of drug-resistance in MTBC strains. Secondly, we recognize the feature weighted model may be biased towards features with higher minor allele frequency. This is also a known existing limitation of RF models using Gini importance measures (Wright *et al.* 2016). This could be improved by aggregating mutations across loci to take into account rarer alleles. Drug-resistance mutations are likely to occur outside of candidate genes and other genomic regions have been implicated in pre-resistance (Torres Ortiz *et al.* 2021). Genome-wide models would promote further discovery and limit the pre-processing required for ML prediction. Finally, the role of putative novel drug-resistance mutations require validation which may be performed using *in silico* and *in vitro* experimentation.

## 5 Conclusion

Previous studies have suggested that ML performance is in part driven by lineage dependency due to confounding from population structure. Evidence presented here confirms that lineage dependency impacts ML predictive performance and interpretability. This study investigated methods to tackle confounding from population structure including stratification, feature selection and FW-RF approaches. FW-RF is a novel approach that is particularly effective at helping us to obtain clearer interpretations of ML prediction, whilst maintaining the power to predict-drug-resistance. However, it is important to consider there may be complex interactions between mutations to bring about drug-resistant phenotypes. Overall, ML approaches have widespread applications to genomic medicine, where genotype-phenotype predictions are being increasingly utilized to gain insight into genomic drivers of disease.

## Supplementary Material

btad428_Supplementary_Data

## Data Availability

The datasets supporting the conclusions of this article are available in the NCBI repository. No new isolates were sequenced during this study. A list of sample and project accession numbers are available in the GitHub repository (https://github.com/NinaMercedes/RF_lineages) along with the code used for analysis and results.

## Declarations

Ethics approval and consent to participate this research conformed to the principles of the Helsinki Declaration. No ethics approval or consent to participate was required, as all data used in the study were already publicly available.
