# Supplementary material for: Feature weighted models to address lineage dependency in drug-resistance prediction from Mycobacterium tuberculosis genome sequences
Source: Bioinformatics. 2023 Jul 10;39(7):btad428. doi: 10.1093/bioinformatics/btad428 (PMC10351970; doi:10.1093/bioinformatics/btad428)
Supplement: btad428_Supplementary_Data [file btad428_supplementary_data.pdf]

Supplementary Material: Feature Weighted Models (FWM) to address lineage dependency in drug-resistance prediction from *Mycobacterium tuberculosis* genome sequences

Nina Billows[1,2], Jody E. Phelan[3], Dong Xia[1], Yonghong Peng[4], Taane G. Clark[3,5], Yu-Mei Chang[1]

Royal Veterinary College, University of London, London, UK [1]

The Alan Turing Institute, British Library, London, UK [2]

Faculty of Infectious and Tropical Diseases, London School of Hygiene & Tropical Medicine, London, UK [3]

Department of Computing and Mathematics, Manchester Metropolitan University, Manchester, UK [4]

Faculty of Epidemiology and Population Health, London School of Hygiene & Tropical Medicine, London, UK [5]

***Correspondence:**

Nina M Billows

nbillows@rvc.ac.uk

List of abbreviations

TB	Tuberculosis
MTBC	Mycobacterium tuberculosis complex
MDR	Multi-drug resistant
XDR	Extremely drug resistant
RF	Random forest
SNPs	Single nucleotide polymorphisms
Indels	Insertions and deletions

Supplementary A

Supplementary Table 1. Methods to reduce lineage-dependency in random forest models applied in this study.

Method	Description
Stratification	Random forest models were trained and tested across all isolates (global), lineages 2 and 4 combined, lineage 2 only and lineage 4 only.
Feature Selection	Lineage-specific variants with a parsimony score <2 were filtered from the dataset. Such variants have a minimum number of state changes equal to one in the tree and would have a weight of 0 in the weighted model.
Feature Weighting	Variants were assigned a feature weight that determined the probability they would be used for splitting in the random forest model. The weight was derived from the minimum number of state changes for each variant in the tree.

Supplementary B

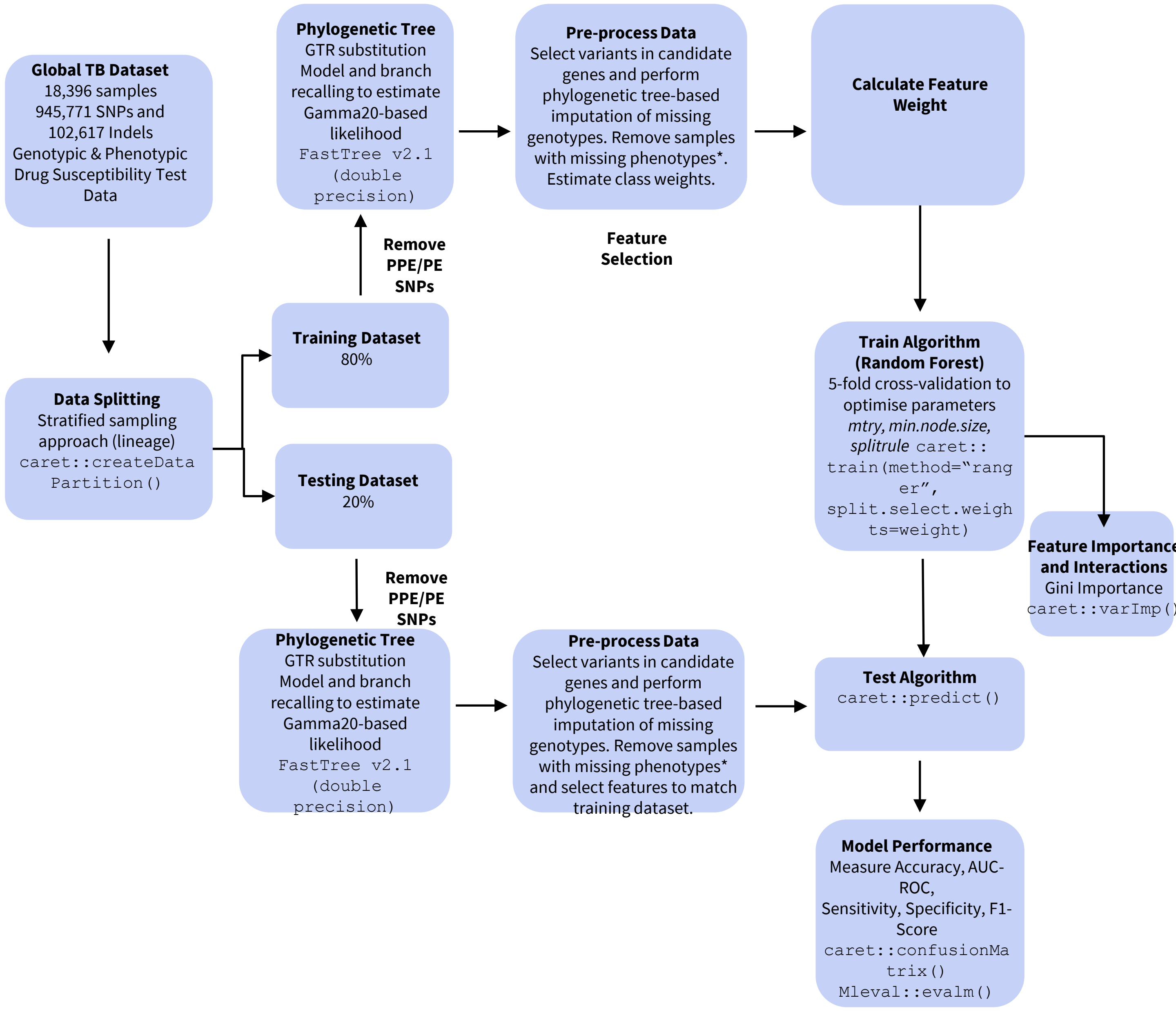

Supplementary Figure 1. Overview of Methods Workflow of methods used in this study. In brief, genotypic and phenotypic information for *18,396 M. tuberculosis* isolates, that had been curated prior to the start of the study, was utilised to predict drug-resistance phenotypes using unweighted and weighted random forest models. Variants in candidate genes were used as features in the model.

Supplementary C

Supplementary Table 2. 29 Candidate genes across 14 drug-resistant Phenotypes used for feature selection.

MDR	RIF	INH	EMB	PZA	OFL	MOX	AMI	CAP	KAN	STM	ETD	CYS	PAS
katG	*rpoB*	*katG*	*embB*	*pncA*	*gyrB*	*gyrB*	*eis*	*tlyA*	*eis*	*gid*	*ethA*	*ald*	*folC*
rpoB	*rpoC*	*inhA*	*embA*	*panD*	*gyrA*	*gyrA*	*rrs*	*rrs*	*rrs*	*rrs*	*inhA*	*alr*	*ribD*
inhA		*ahpC*	*embC*	*rpsA*						*rpsL*	*ethR*		*thyA*
ahpC		*fabG1*	*embR*								*fabG1*		*thyX*
fabG1		*kasA*											
kasA													
rpoC													

Supplementary D

Supplementary Table 3. Performance of global, combined and lineage-specific random forest models predicting 14 drug-resistant phenotypes in terms of AUC-ROC, Sensitivity, Specificity and F1 Score.

Drug	Lineage	Resistant (N)	Susceptible (N)	Resistant (%)	AUC	AUC (95% CI)	Sensitivity	Sensitivity (95% CI)	Specificity	Specificity (95% CI)	F1
	Global	3963	10382	27.63	0.96	0.96-0.96	0.878	0.87-0.89	0.951	0.95-0.95	0.875
	Lineage 2	1895	1629	53.77	0.96	0.95-0.97	0.885	0.87-0.9	0.923	0.91-0.94	0.908
**MDR**	Lineage 4	1549	5391	22.32	0.98	0.98-0.98	0.879	0.86-0.89	0.98	0.98-0.98	0.901
	Lineage 2 & 4	3444	7020	32.91	0.97	0.97-0.97	0.869	0.86-0.88	0.961	0.96-0.96	0.892
	Global	4324	10149	29.88	0.96	0.96-0.96	0.878	0.87-0.89	0.951	0.95-0.95	0.875
	Lineage 2	2081	1544	57.41	0.97	0.96-0.98	0.88	0.87-0.89	0.936	0.92-0.95	0.913
**RIF**	Lineage 4	1698	5265	24.39	0.97	0.96-0.98	0.844	0.83-0.86	0.98	0.98-0.98	0.885
	Lineage 2 & 4	3779	6809	35.69	0.97	0.97-0.97	0.859	0.85-0.87	0.963	0.96-0.97	0.892
	Global	4957	9357	34.63	0.93	0.92-0.94	0.788	0.78-0.8	0.965	0.96-0.97	0.85
	Lineage 2	2119	1394	60.32	0.97	0.96-0.98	0.853	0.84-0.87	0.978	0.97-0.98	0.914
**INH**	Lineage 4	2034	4904	29.32	0.95	0.94-0.96	0.814	0.8-0.83	0.992	0.99-0.99	0.888
	Lineage 2 & 4	4153	6298	39.74	0.95	0.95-0.95	0.814	0.8-0.83	0.977	0.97-0.98	0.881
	Global	2503	10768	18.86	0.92	0.91-0.93	0.862	0.85-0.88	0.877	0.87-0.88	0.721
	Lineage 2	1217	2277	34.83	0.88	0.87-0.89	0.855	0.83-0.87	0.769	0.75-0.79	0.748
**EMB**	Lineage 4	1027	5170	16.57	0.95	0.94-0.96	0.847	0.82-0.87	0.93	0.92-0.94	0.77
	Lineage 2 & 4	2244	7447	23.16	0.92	0.91-0.93	0.859	0.84-0.87	0.87	0.86-0.88	0.75
	Global	1853	8738	17.5	0.93	0.92-0.94	0.838	0.82-0.85	0.889	0.88-0.9	0.71
	Lineage 2	773	1490	34.16	0.87	0.85-0.89	0.825	0.8-0.85	0.775	0.75-0.8	0.731
**PZA**	Lineage 4	829	4546	15.42	0.95	0.94-0.96	0.866	0.84-0.89	0.934	0.93-0.94	0.778
	Lineage 2 & 4	1602	6036	20.97	0.93	0.92-0.94	0.86	0.84-0.88	0.863	0.85-0.87	0.723
	Global	593	2143	21.67	0.89	0.87-0.91	0.74	0.7-0.77	0.936	0.92-0.95	0.751
	Lineage 2	351	1187	22.82	0.89	0.87-0.91	0.701	0.65-0.75	0.95	0.94-0.96	0.75
**OFL**	Lineage 4	203	847	19.33	0.89	0.86-0.92	0.704	0.64-0.76	0.926	0.91-0.94	0.699
	Lineage 2 & 4	554	2034	21.41	0.89	0.87-0.91	0.74	0.7-0.77	0.932	0.92-0.94	0.743
	Global	245	1231	16.6	0.85	0.82-0.88	0.78	0.72-0.83	0.763	0.74-0.79	0.525
	Lineage 2	183	613	22.99	0.86	0.83-0.89	0.782	0.72-0.83	0.756	0.73-0.78	0.53
**MOX**	Lineage 4	55	525	9.48	0.86	0.8-0.92	0.564	0.43-0.69	0.926	0.9-0.95	0.496
	Lineage 2 & 4	238	1138	17.3	0.86	0.83-0.89	0.782	0.72-0.83	0.756	0.73-0.78	0.53
	Global	315	1835	14.65	0.91	0.89-0.93	0.727	0.68-0.77	0.955	0.94-0.96	0.73
	Lineage 2	213	879	19.51	0.9	0.87-0.93	0.737	0.67-0.79	0.97	0.96-0.98	0.793
**AMI**	Lineage 4	96	744	11.43	0.86	0.81-0.91	0.688	0.59-0.77	0.937	0.92-0.95	0.632
	Lineage 2 & 4	309	1623	15.99	0.9	0.88-0.92	0.718	0.67-0.77	0.961	0.95-0.97	0.746
	Global	356	1992	15.16	0.89	0.87-0.91	0.697	0.65-0.74	0.964	0.95-0.97	0.734
	Lineage 2	223	1001	18.22	0.86	0.83-0.89	0.664	0.6-0.72	0.969	0.96-0.98	0.736
**CAP**	Lineage 4	131	807	13.97	0.87	0.83-0.91	0.649	0.56-0.73	0.963	0.95-0.97	0.691
	Lineage 2 & 4	354	1808	16.37	0.88	0.86-0.9	0.678	0.63-0.72	0.966	0.96-0.97	0.732
	Global	493	1902	20.58	0.94	0.93-0.95	0.787	0.75-0.82	0.97	0.96-0.98	0.826
	Lineage 2	180	894	16.76	0.92	0.89-0.95	0.75	0.68-0.81	0.98	0.97-0.99	0.811
**KAN**	Lineage 4	304	775	28.17	0.96	0.94-0.98	0.839	0.79-0.88	0.978	0.97-0.99	0.885
	Lineage 2 & 4	484	1669	22.48	0.94	0.93-0.95	0.771	0.73-0.81	0.974	0.96-0.98	0.828
	Global	2033	3914	34.19	0.91	0.9-0.92	0.818	0.8-0.83	0.888	0.88-0.9	0.804
	Lineage 2	1361	1070	55.99	0.94	0.93-0.95	0.868	0.85-0.89	0.902	0.88-0.92	0.893
**STM**	Lineage 4	581	1991	22.59	0.9	0.88-0.92	0.737	0.7-0.77	0.914	0.9-0.93	0.725
	Lineage 2 & 4	1942	3061	38.82	0.92	0.91-0.93	0.805	0.79-0.82	0.911	0.9-0.92	0.828
	Global	86	747	10.32	0.78	0.72-0.84	0.419	0.32-0.52	0.902	0.88-0.92	0.369
	Lineage 2	65	497	11.57	0.79	0.72-0.86	0.369	0.26-0.49	0.909	0.88-0.93	0.358
**CYS**	Lineage 4	21	243	7.95	0.72	0.59-0.85	0.524	0.32-0.72	0.877	0.83-0.91	0.355
	Lineage 2 & 4	86	740	10.41	0.77	0.71-0.83	0.407	0.31-0.51	0.899	0.87-0.92	0.357
	Global	543	1188	31.37	0.79	0.77-0.81	0.495	0.45-0.54	0.879	0.86-0.9	0.563
	Lineage 2	328	800	29.08	0.74	0.71-0.77	0.549	0.49-0.6	0.79	0.76-0.82	0.533
**ETD**	Lineage 4	214	367	36.83	0.86	0.83-0.89	0.696	0.63-0.75	0.869	0.83-0.9	0.725
	Lineage 2 & 4	542	1167	31.71	0.78	0.75-0.81	0.478	0.44-0.52	0.882	0.86-0.9	0.552
	Global	68	845	7.45	0.71	0.64-0.78	0.368	0.26-0.49	0.91	0.89-0.93	0.296
	Lineage 2	50	578	7.96	0.72	0.64-0.8	0.4	0.28-0.54	0.92	0.9-0.94	0.345
**PAS**	Lineage 4	17	262	6.09	0.6	0.45-0.75	0.353	0.17-0.59	0.836	0.79-0.88	0.182
	Lineage 2 & 4	67	840	7.39	0.71	0.64-0.78	0.313	0.22-0.43	0.929	0.91-0.94	0.284

Supplementary E

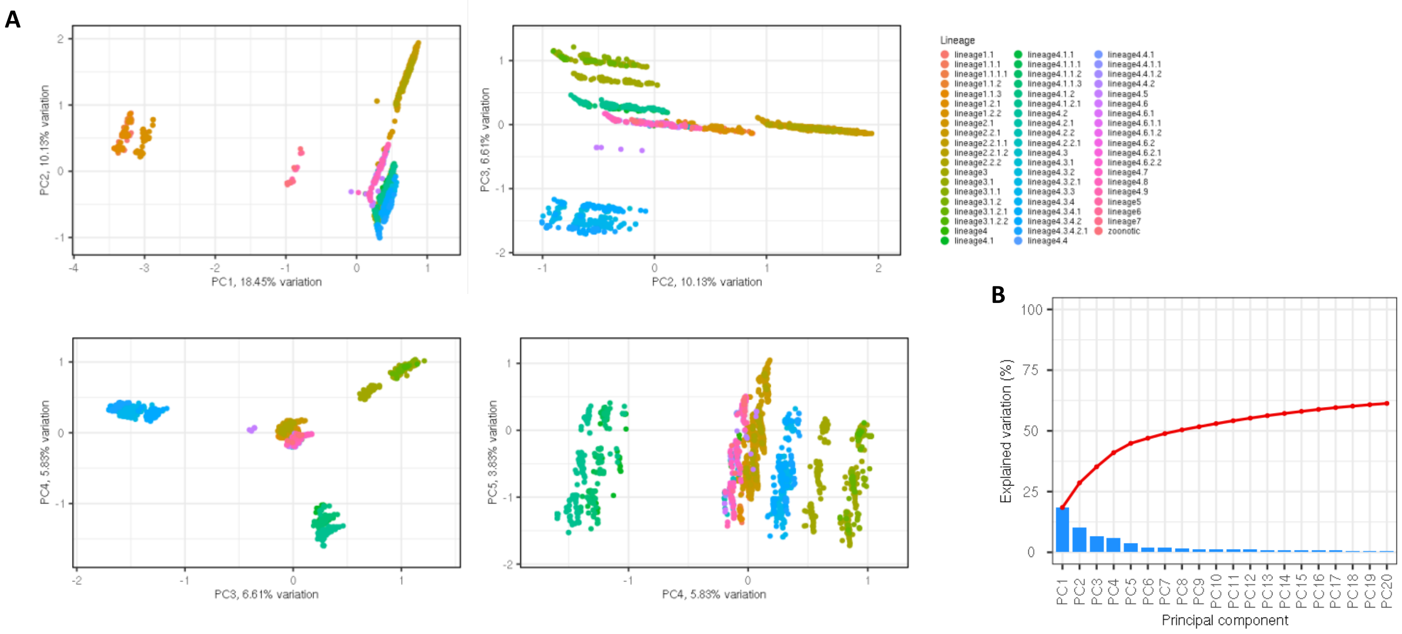

Supplementary Figure 2. Principal component analysis of global training dataset. The first 5 principal components are plotted and are coloured according to sub-lineage (A). A scree plot showing the percentage of variation accounted for by the first 20 principal components is shown (B)

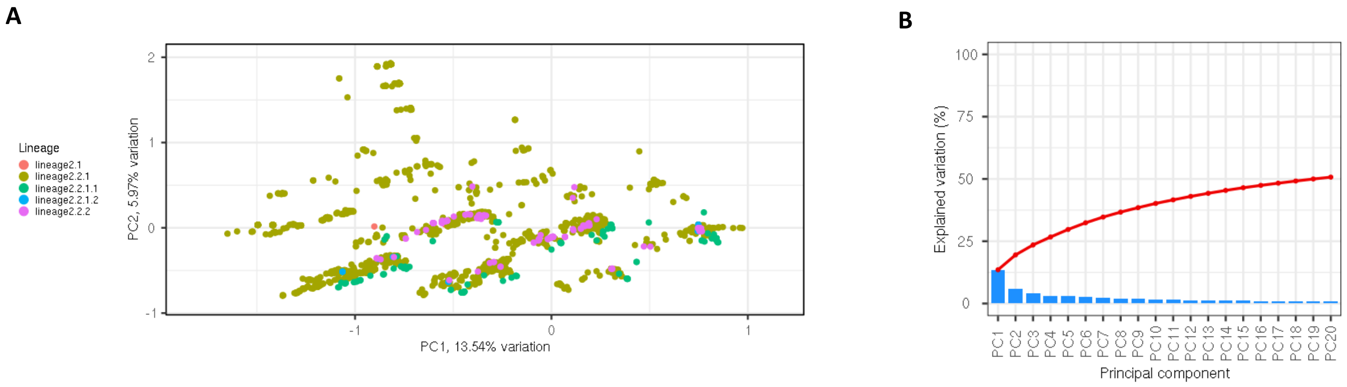

Supplementary Figure 3. Principal component analysis of lineage 2 training dataset. The first 5 principal components are plotted and are coloured according to sub-lineage (A). A scree plot showing the percentage of variation accounted for by the first 20 principal components is shown (B)

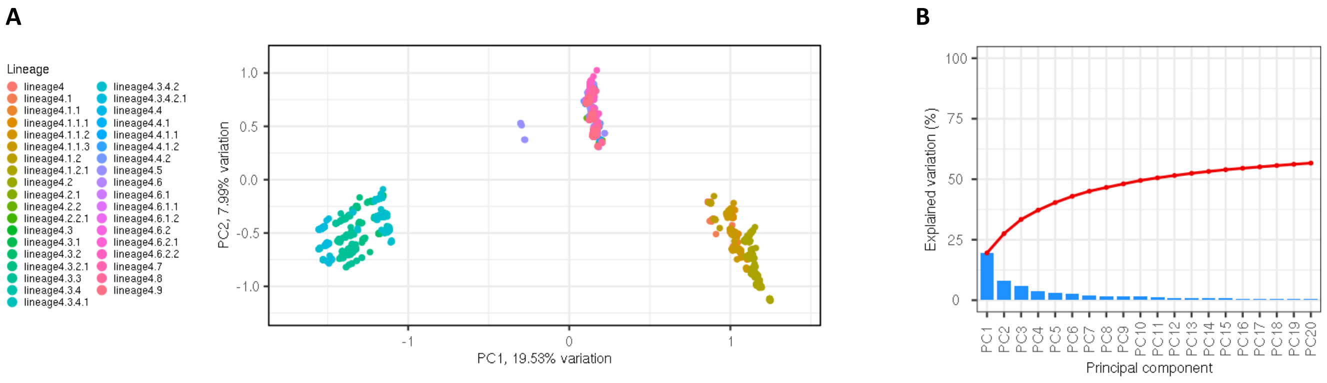

Supplementary Figure 4. Principal component analysis of lineage 4 training dataset. The first 5 principal components are plotted and are coloured according to sub-lineage (A). A scree plot showing the percentage of variation accounted for by the first 20 principal components is shown (B)

Supplementary F

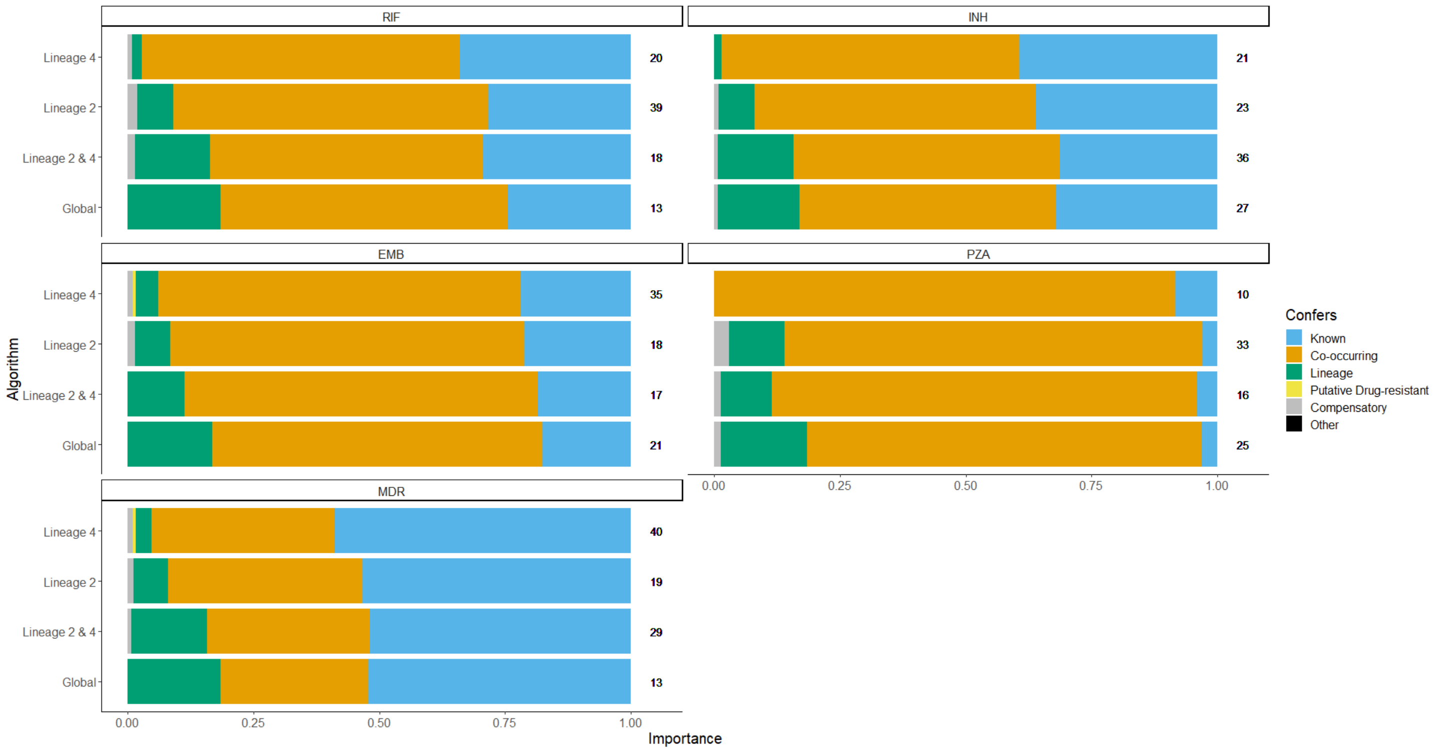

Supplementary Figure 5. Interpretability of global, combined and lineage-specific random forest models predicting resistance to first-line drugs and MDR-TB phenotype. The proportion of variants that are classified as a known drug-resistance mutation (blue), co-occurring mutation (orange), lineage-specific mutation (green), putative drug-resistant (yellow) and other are shown for the global, combined and lineage-specific. Threshold for the number of 'most important variants' are indicated next to the bar.

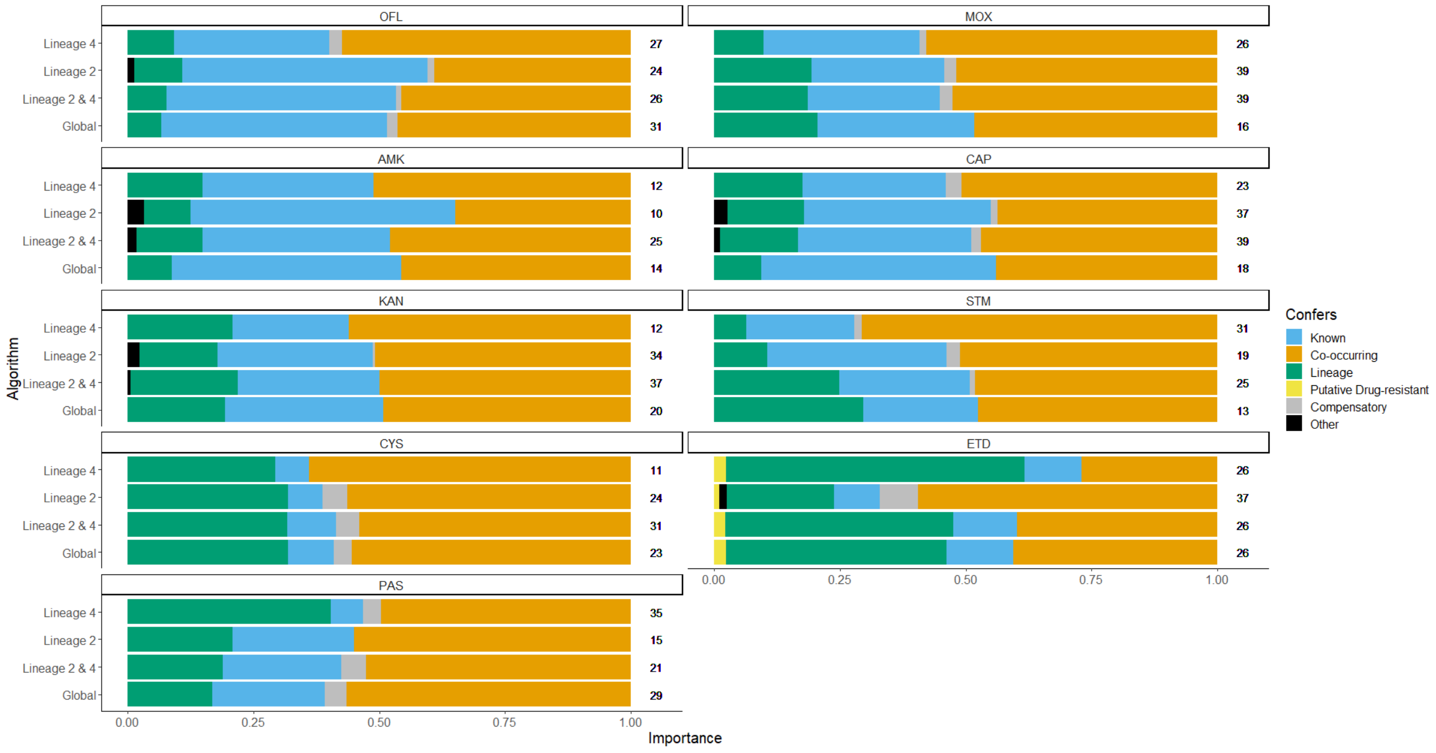

Supplementary Figure 6. Interpretability of global, combined and lineage-specific random forest models predicting resistance to second-line drugs. The proportion of variants that are classified as a known drug-resistance mutation (blue), co-occurring mutation (orange), lineage-specific mutation (green), putative drug-resistant (yellow) and other are shown for the global, combined and lineage-specific models. Threshold for the number of 'most important variants' are indicated next to the bar.

Supplementary G

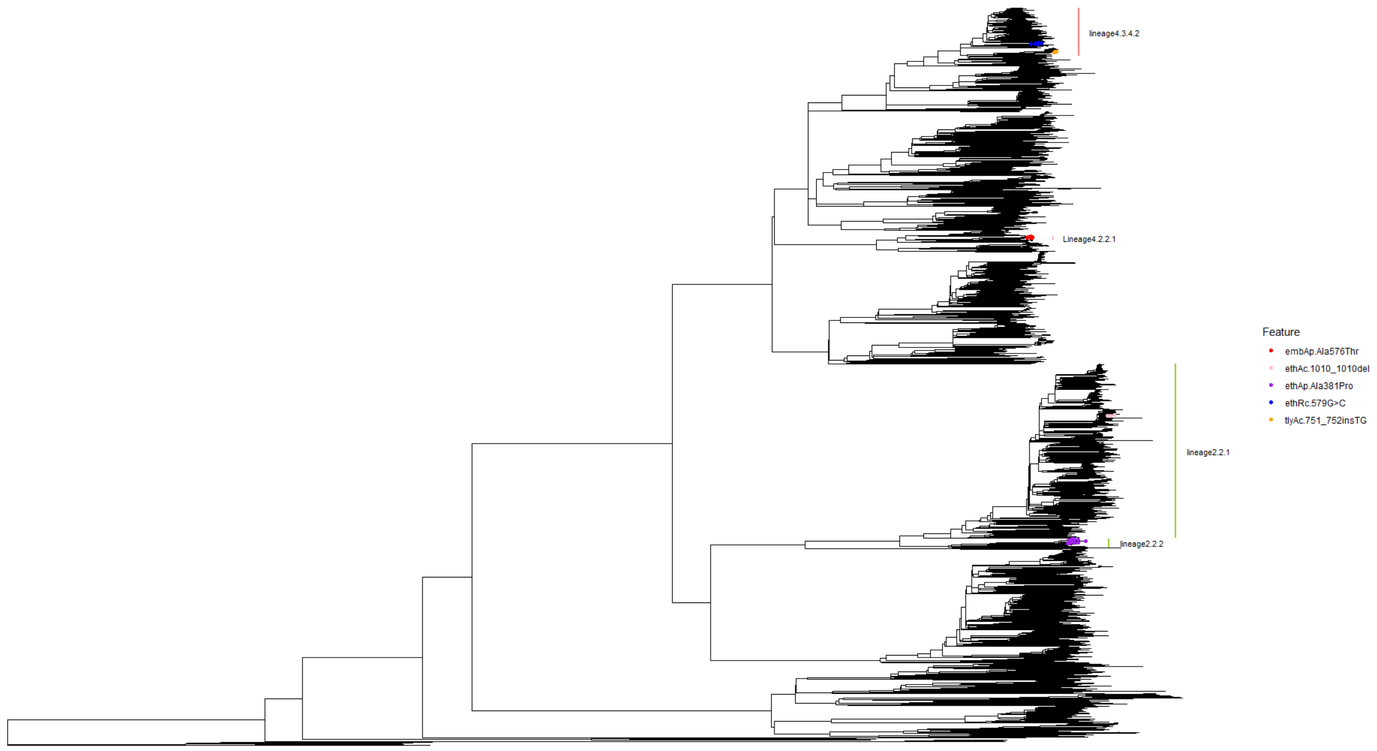

Supplementary Figure 7. Phylogenetic distribution of variants of interest across the training dataset. Maximum likelihood tree of the training dataset is shown rooted on an *M. canetti* isolate. Tips are annotated to show the distribution of lineage-specific mutations thought to play a role in drug-resistance and are coloured according to each feature. Sub-lineages where such mutations are found are labelled accordingly.

Supplementary H

Supplementary Table 5. Performance of unweighted and feature weighted random forest models predicting 14 drug-resistant phenotypes in terms of AUC-ROC, Sensitivity, Specificity and F1 Score.

Drug	Algorithm	AUC	AUC (95% CI)	Sensitivity	Sensitivity (95% CI)	Specificity	Specificity (95% CI)	F1
	Unweighted RF	0.96	0.96-0.96	0.878	0.87-0.89	0.951	0.95-0.95	0.875
	Unweighted RF (Parsimony Score <2)	0.98	0.98-0.98	0.889	0.88-0.9	0.97	0.97-0.97	0.904
**MDR**	Weighted RF (Parsimony Score)	0.97	0.97-0.97	0.929	0.92-0.94	0.953	0.95-0.96	0.906
	Unweighted RF	0.96	0.96-0.96	0.878	0.87-0.89	0.951	0.95-0.95	0.875
	Unweighted RF (Parsimony Score <2)	0.97	0.97-0.97	0.885	0.87-0.89	0.974	0.97-0.98	0.91
**RIF**	Weighted RF (Parsimony Score)	0.98	0.98-0.98	0.929	0.92-0.94	0.961	0.96-0.96	0.915
	Unweighted RF	0.93	0.92-0.94	0.788	0.78-0.8	0.965	0.96-0.97	0.85
	Unweighted RF (Parsimony Score <2)	0.95	0.95-0.95	0.846	0.84-0.86	0.988	0.99-0.99	0.906
**INH**	Weighted RF (Parsimony Score)	0.94	0.94-0.94	0.866	0.86-0.87	0.985	0.98-0.99	0.914
	Unweighted RF	0.92	0.91-0.93	0.862	0.85-0.88	0.877	0.87-0.88	0.721
	Unweighted RF (Parsimony Score <2)	0.94	0.93-0.95	0.886	0.87-0.9	0.879	0.87-0.89	0.736
**EMB**	Weighted RF (Parsimony Score)	0.94	0.93-0.95	0.906	0.89-0.92	0.868	0.86-0.87	0.732
	Unweighted RF	0.93	0.92-0.94	0.838	0.82-0.85	0.889	0.88-0.9	0.71
	Unweighted RF (Parsimony Score <2)	0.94	0.93-0.95	0.882	0.87-0.9	0.87	0.86-0.88	0.707
**PZA**	Weighted RF (Parsimony Score)	0.93	0.92-0.94	0.851	0.83-0.87	0.86	0.85-0.87	0.678
	Unweighted RF	0.89	0.87-0.91	0.74	0.7-0.77	0.936	0.92-0.95	0.751
	Unweighted RF (Parsimony Score <2)	0.9	0.88-0.92	0.757	0.72-0.79	0.927	0.91-0.94	0.749
**OFL**	Weighted RF (Parsimony Score)	0.91	0.89-0.93	0.728	0.69-0.76	0.964	0.96-0.97	0.784
	Unweighted RF	0.85	0.82-0.88	0.78	0.72-0.83	0.763	0.74-0.79	0.525
	Unweighted RF (Parsimony Score <2)	0.88	0.85-0.91	0.784	0.73-0.83	0.814	0.79-0.83	0.577
**MOX**	Weighted RF (Parsimony Score)	0.87	0.84-0.9	0.686	0.63-0.74	0.898	0.88-0.91	0.623
	Unweighted RF	0.91	0.89-0.93	0.727	0.68-0.77	0.955	0.94-0.96	0.73
	Unweighted RF (Parsimony Score <2)	0.92	0.9-0.94	0.756	0.71-0.8	0.96	0.95-0.97	0.759
**AMI**	Weighted RF (Parsimony Score)	0.9	0.88-0.92	0.759	0.71-0.8	0.948	0.94-0.96	0.735
	Unweighted RF	0.89	0.87-0.91	0.697	0.65-0.74	0.964	0.95-0.97	0.734
	Unweighted RF (Parsimony Score <2)	0.88	0.86-0.9	0.711	0.66-0.76	0.964	0.95-0.97	0.743
**CAP**	Weighted RF (Parsimony Score)	0.86	0.83-0.89	0.702	0.65-0.75	0.952	0.94-0.96	0.713
	Unweighted RF	0.94	0.93-0.95	0.787	0.75-0.82	0.97	0.96-0.98	0.826
	Unweighted RF (Parsimony Score <2)	0.95	0.94-0.96	0.809	0.77-0.84	0.986	0.98-0.99	0.868
**KAN**	Weighted RF (Parsimony Score)	0.94	0.93-0.95	0.828	0.79-0.86	0.968	0.96-0.97	0.848
	Unweighted RF	0.91	0.9-0.92	0.818	0.8-0.83	0.888	0.88-0.9	0.804
	Unweighted RF (Parsimony Score <2)	0.93	0.92-0.94	0.846	0.83-0.86	0.91	0.9-0.92	0.838
**STM**	Weighted RF (Parsimony Score)	0.94	0.93-0.95	0.841	0.82-0.86	0.927	0.92-0.94	0.849
	Unweighted RF	0.78	0.72-0.84	0.419	0.32-0.52	0.902	0.88-0.92	0.369
	Unweighted RF (Parsimony Score <2)	0.71	0.65-0.77	0.453	0.35-0.56	0.897	0.87-0.92	0.386
**CYS**	Weighted RF (Parsimony Score)	0.67	0.6-0.74	0.477	0.37-0.58	0.842	0.81-0.87	0.335
	Unweighted RF	0.79	0.77-0.81	0.495	0.45-0.54	0.879	0.86-0.9	0.563
	Unweighted RF (Parsimony Score <2)	0.78	0.75-0.81	0.532	0.49-0.57	0.839	0.82-0.86	0.565
**ETD**	Weighted RF (Parsimony Score)	0.77	0.74-0.8	0.584	0.54-0.62	0.818	0.8-0.84	0.589
	Unweighted RF	0.71	0.64-0.78	0.368	0.26-0.49	0.91	0.89-0.93	0.296
	Unweighted RF (Parsimony Score <2)	0.7	0.63-0.77	0.426	0.32-0.54	0.909	0.89-0.93	0.333
**PAS**	Weighted RF (Parsimony Score)	0.65	0.58-0.72	0.338	0.24-0.46	0.789	0.76-0.82	0.171

Supplementary I

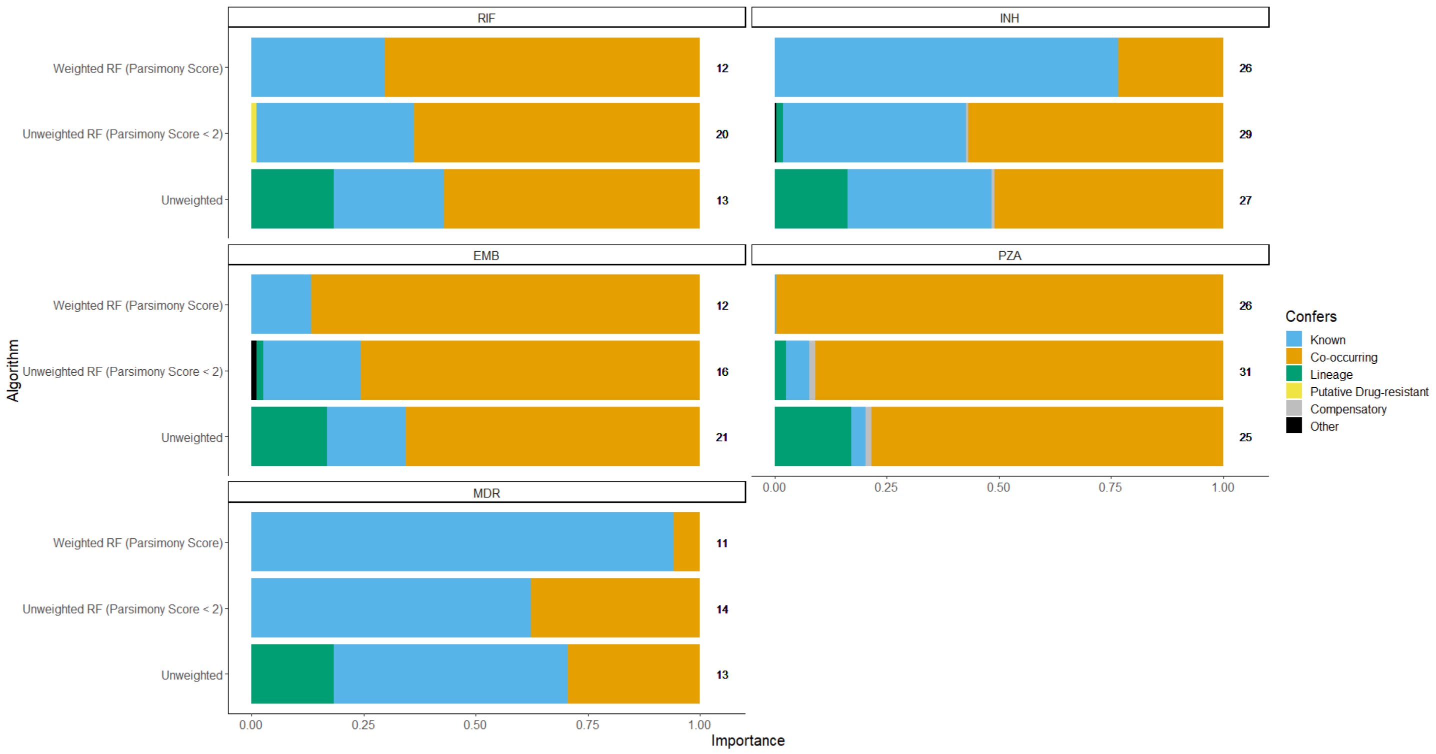

Supplementary Figure 8. Interpretability of unweighted and feature weighted random forest models predicting resistance to first-line drugs and MDR-TB phenotype. The proportion of variants that are classified as a known drug-resistance mutation (blue), co-occurring mutation (orange), lineage-specific mutation (green), putative drug-resistant (yellow) and other are shown for the unweighted and weighted models. Threshold for the number of 'most important variants' are indicated next to the bar.

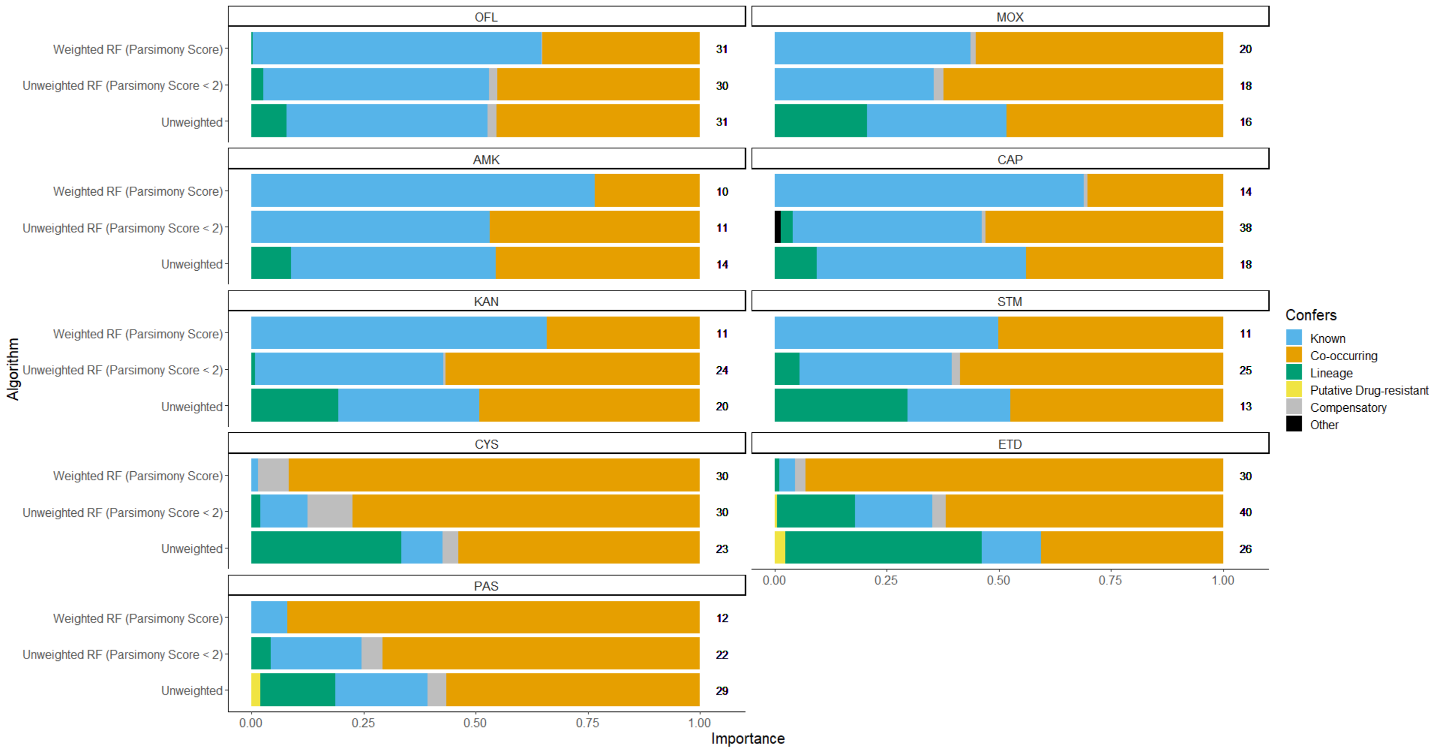

Supplementary Figure 9. Interpretability of unweighted and feature weighted random forest models predicting resistance to second-line drugs. The proportion of variants that are classified as a known drug-resistance mutation (blue), co-occurring mutation (orange), lineage-specific mutation (green), putative drug-resistant (yellow) and other are shown for the unweighted and weighted models. Threshold for the number of 'most important variants' are indicated next to the bar.

Supplementary J

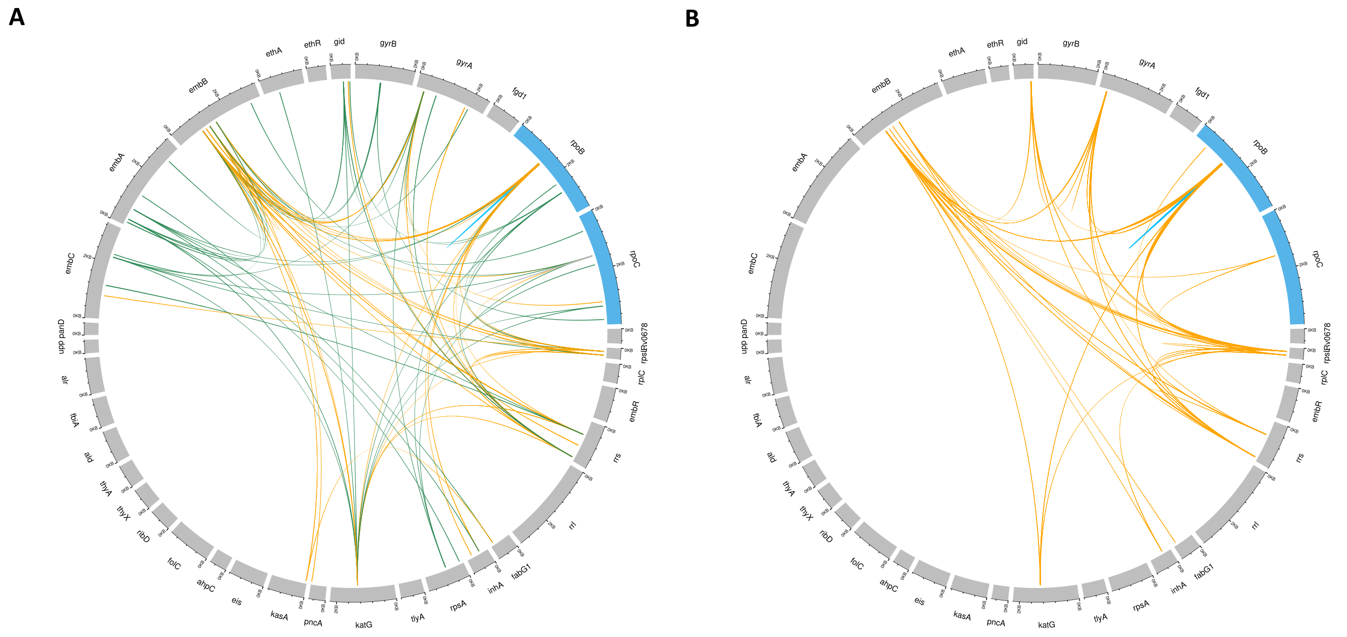

Supplementary Figure 10. Most frequent interactions (top 1%) observed across 1,000 trees in random forest model for rifampicin-resistance prediction. A) Most frequent variant-variant interactions in the unweighted model. B) Most frequent variant-variant interactions in the weighted model. Genes known to contain rifampicin-resistance mutations are highlighted in blue. Interactions are classified as a known drug-resistance interaction (blue), co-occurring interaction (orange) and lineage interaction (green).

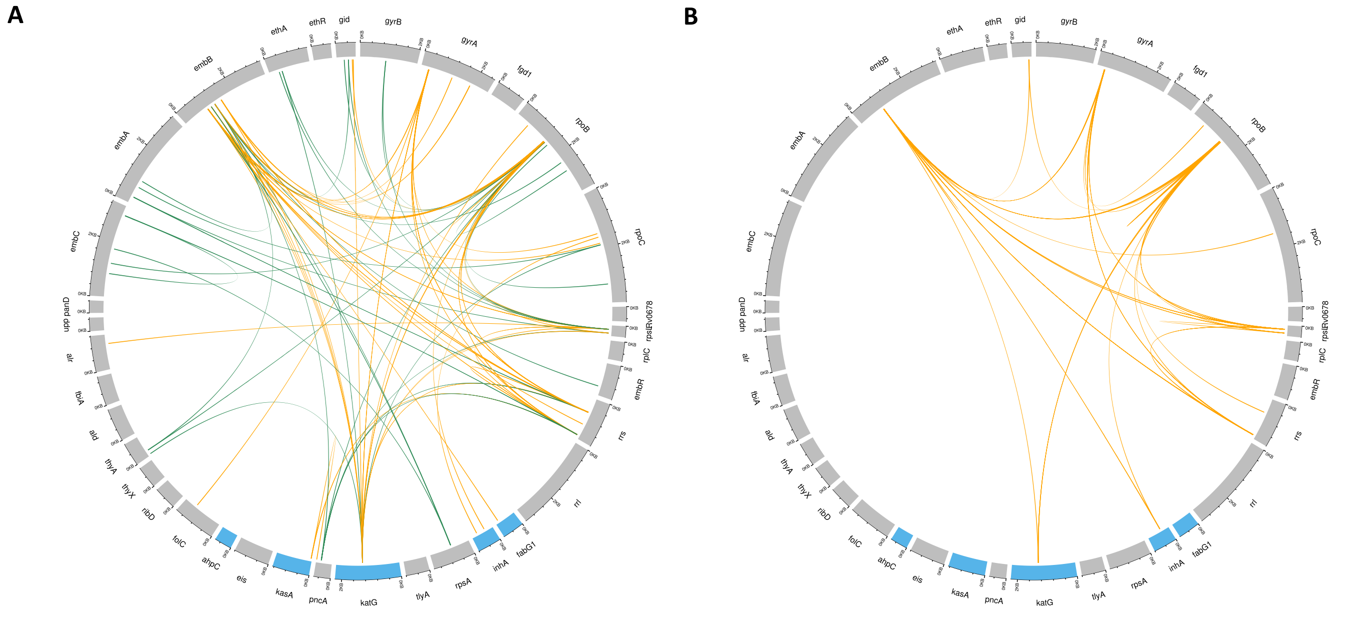

Supplementary Figure 11. Most frequent interactions (top 1%) observed across 1,000 trees in random forest model for isoniazid-resistance prediction. A) Most frequent variant-variant interactions in the unweighted model. B) Most frequent variant-variant interactions in the weighted model. Genes known to contain isoniazid-resistance mutations are highlighted in blue. Interactions are classified as a known drug-resistance interaction (blue), co-occurring interaction (orange) and lineage interaction (green).

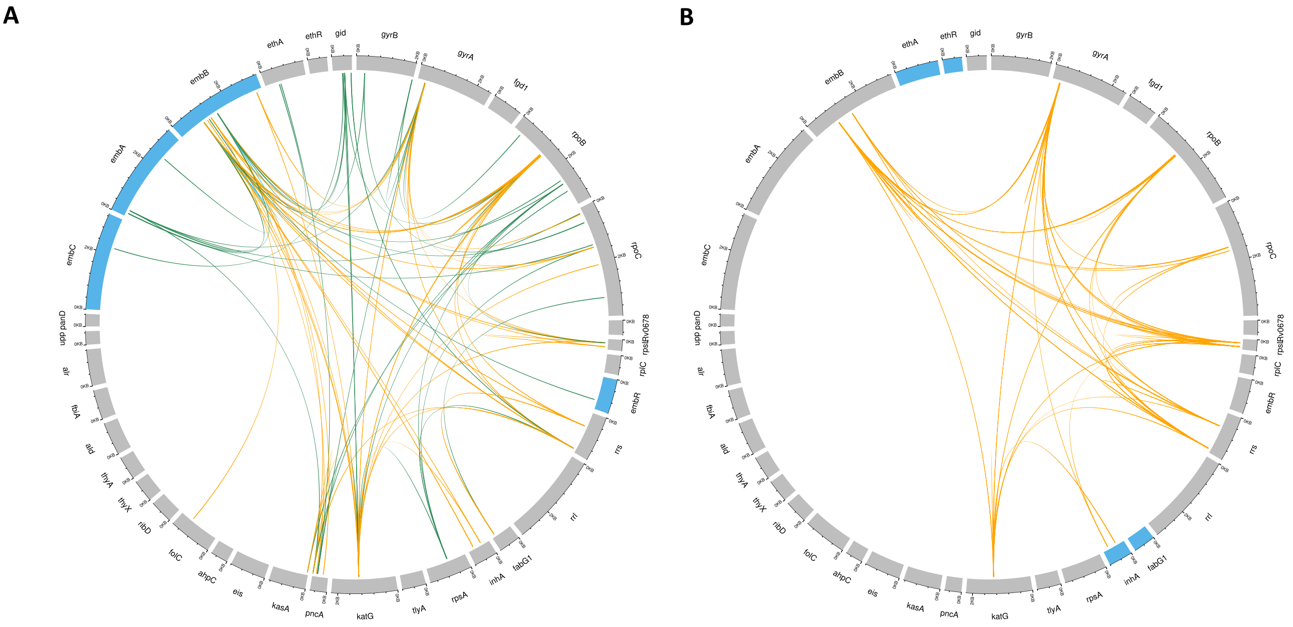

Supplementary Figure 12. Most frequent interactions (top 1%) observed across 1,000 trees in random forest model for ethambutol-resistance prediction. A) Most frequent variant-variant interactions in the unweighted model. B) Most frequent variant-variant interactions in the weighted model. Genes known to contain ethambutol-resistance mutations are highlighted in blue. Interactions are classified as a known drug-resistance interaction (blue), co-occurring interaction (orange) and lineage interaction (green).

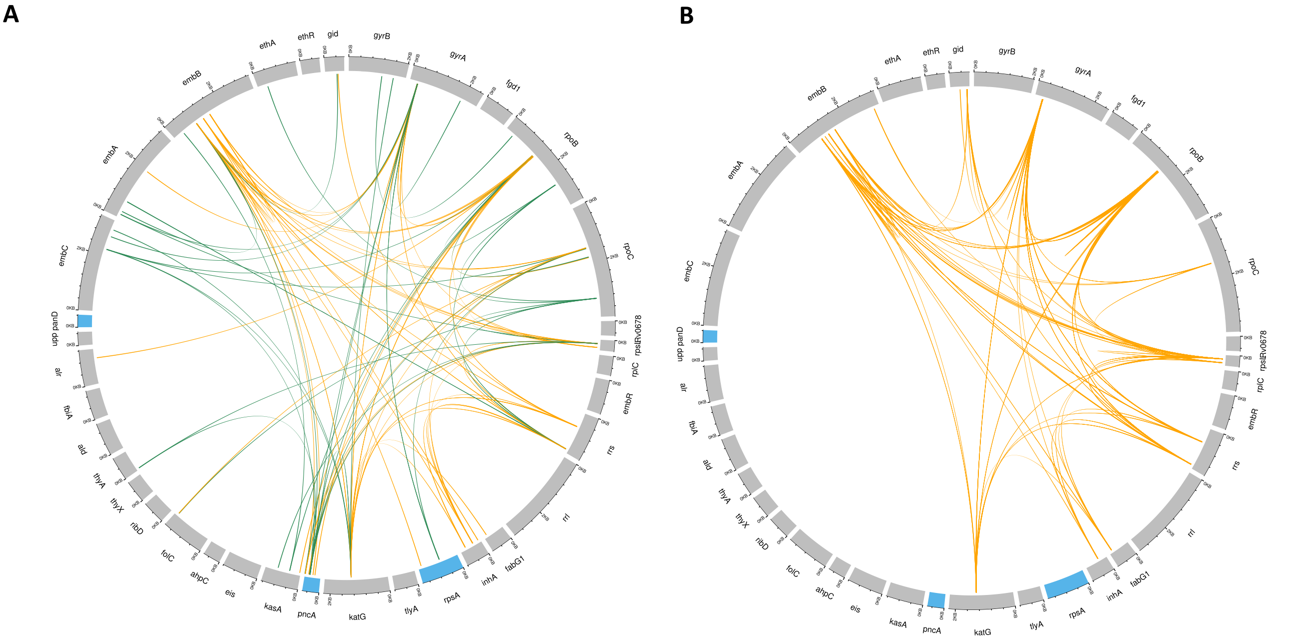

Supplementary Figure 13. Most frequent interactions (top 1%) observed across 1,000 trees in random forest model for pyrazinamide-resistance prediction. A) Most frequent variant-variant interactions in the unweighted model. B) Most frequent variant-variant interactions in the weighted model. Genes known to contain pyrazinamide-resistance mutations are highlighted in blue. Interactions are classified as a known drug-resistance interaction (blue), co-occurring interaction (orange) and lineage interaction (green).

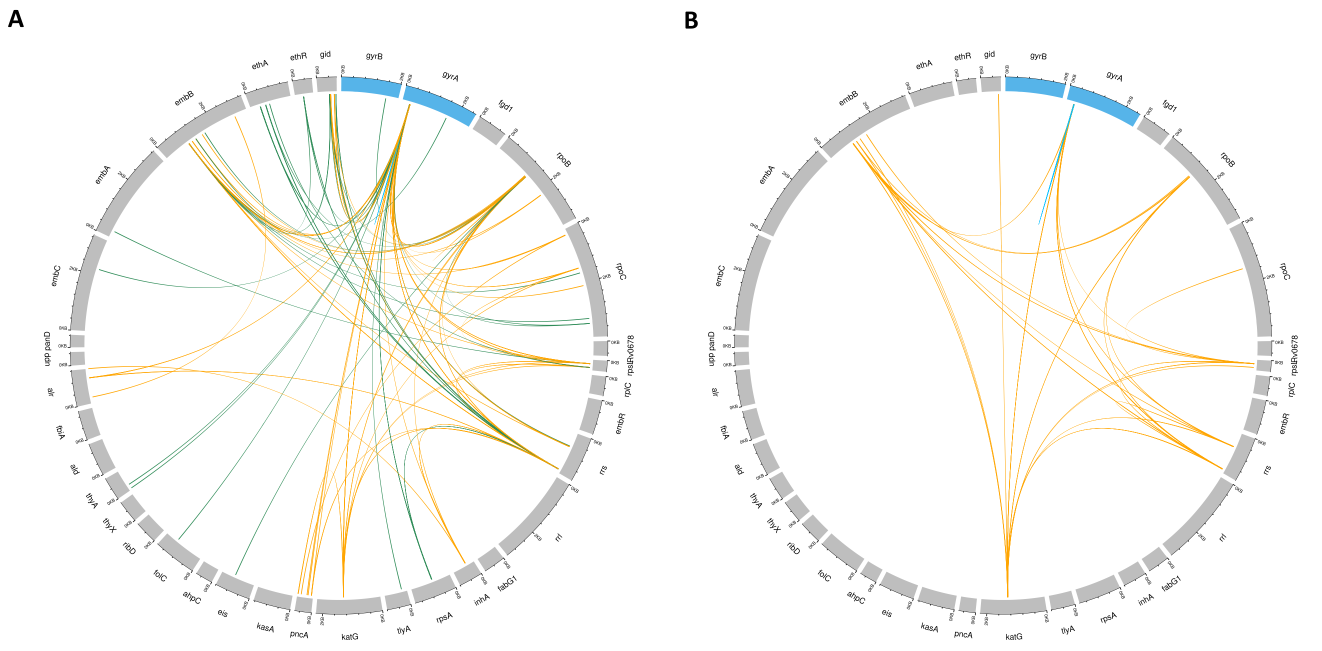

Supplementary Figure 14. Most frequent interactions (top 1%) observed across 1,000 trees in random forest model for ofloxacin-resistance prediction. A) Most frequent variant-variant interactions in the unweighted model. B) Most frequent variant-variant interactions in the weighted model. Genes known to contain ofloxacin-resistance mutations are highlighted in blue. Interactions are classified as a known drug-resistance interaction (blue), co-occurring interaction (orange) and lineage interaction (green).

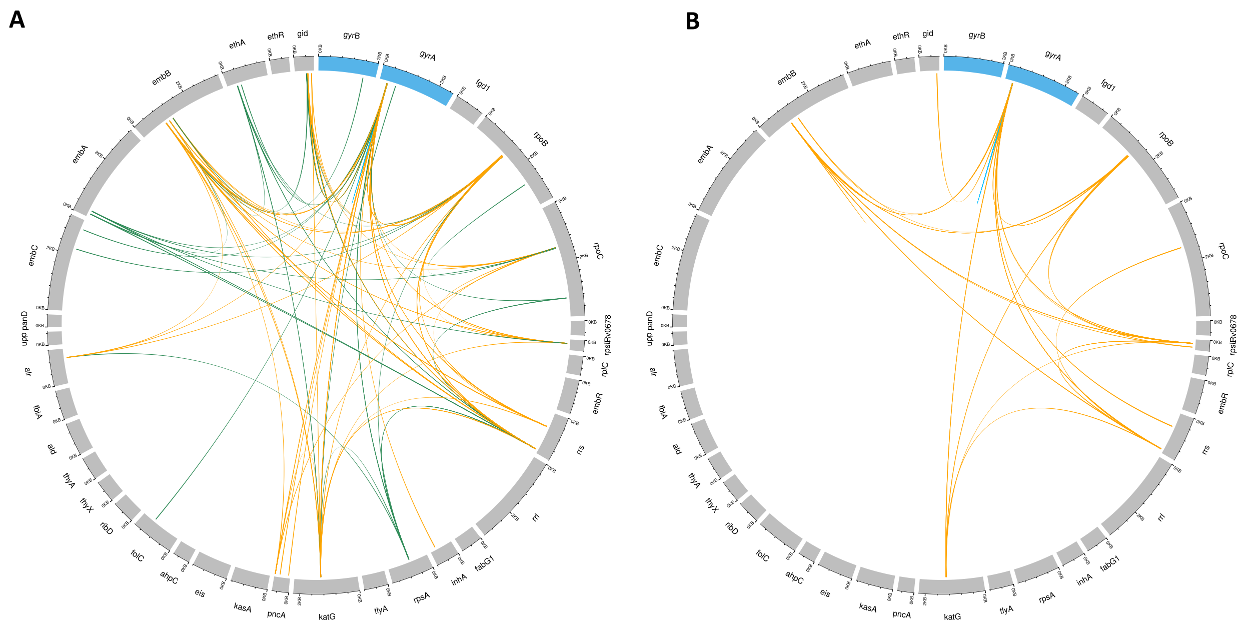

Supplementary Figure 15. Most frequent interactions (top 1%) observed across 1,000 trees in random forest model for moxifloxacin-resistance prediction. A) Most frequent variant-variant interactions in the unweighted model. B) Most frequent variant-variant interactions in the weighted model. Genes known to contain moxifloxacin-resistance mutations are highlighted in blue. Interactions are classified as a known drug-resistance interaction (blue), co-occurring interaction (orange) and lineage interaction (green).

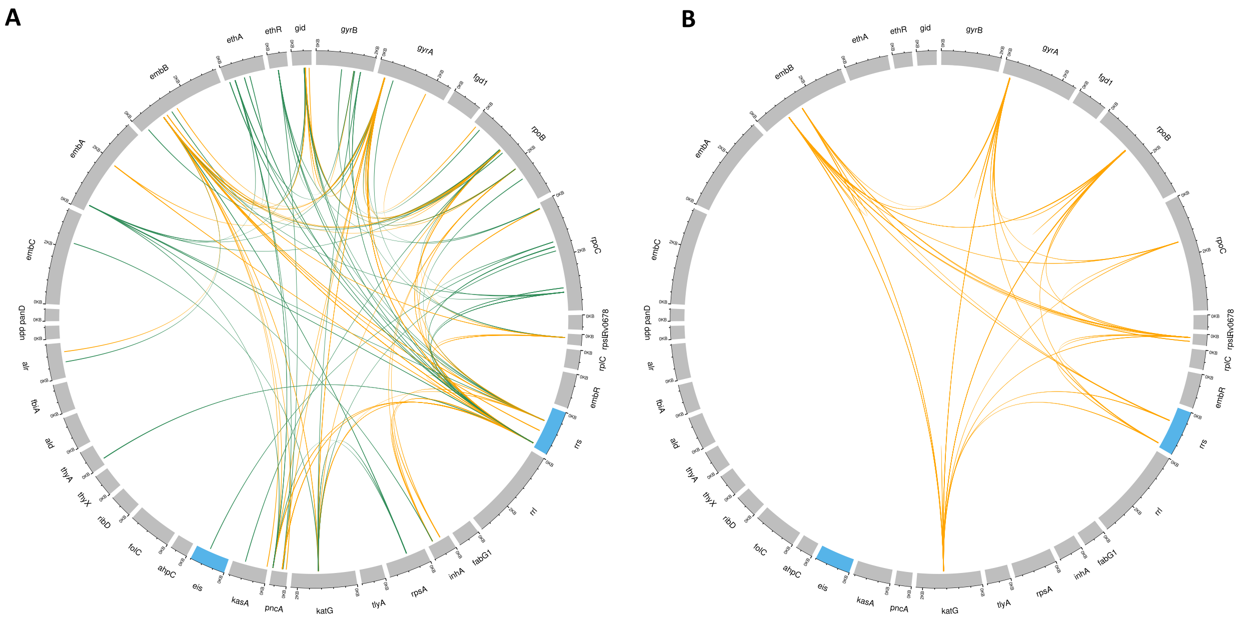

Supplementary Figure 16. Most frequent interactions (top 1%) observed across 1,000 trees in random forest model for amikacin-resistance prediction. A) Most frequent variant-variant interactions in the unweighted model. B) Most frequent variant-variant interactions in the weighted model. Genes known to contain amikacin-resistance mutations are highlighted in blue. Interactions are classified as a known drug-resistance interaction (blue), co-occurring interaction (orange) and lineage interaction (green).

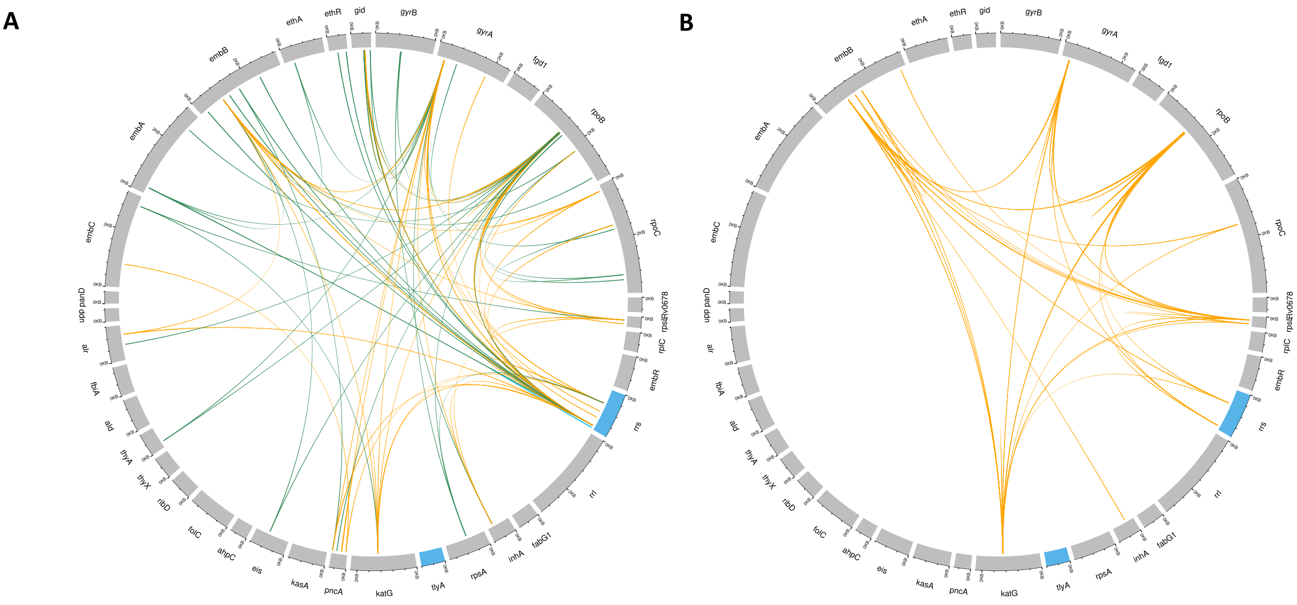

Supplementary Figure 17. Most frequent interactions (top 1%) observed across 1,000 trees in random forest model for capreomycin-resistance prediction. A) Most frequent variant-variant interactions in the unweighted model. B) Most frequent variant-variant interactions in the weighted model. Genes known to contain capreomycin-resistance mutations are highlighted in blue. Interactions are classified as a known drug-resistance interaction (blue), co-occurring interaction (orange) and lineage interaction (green).

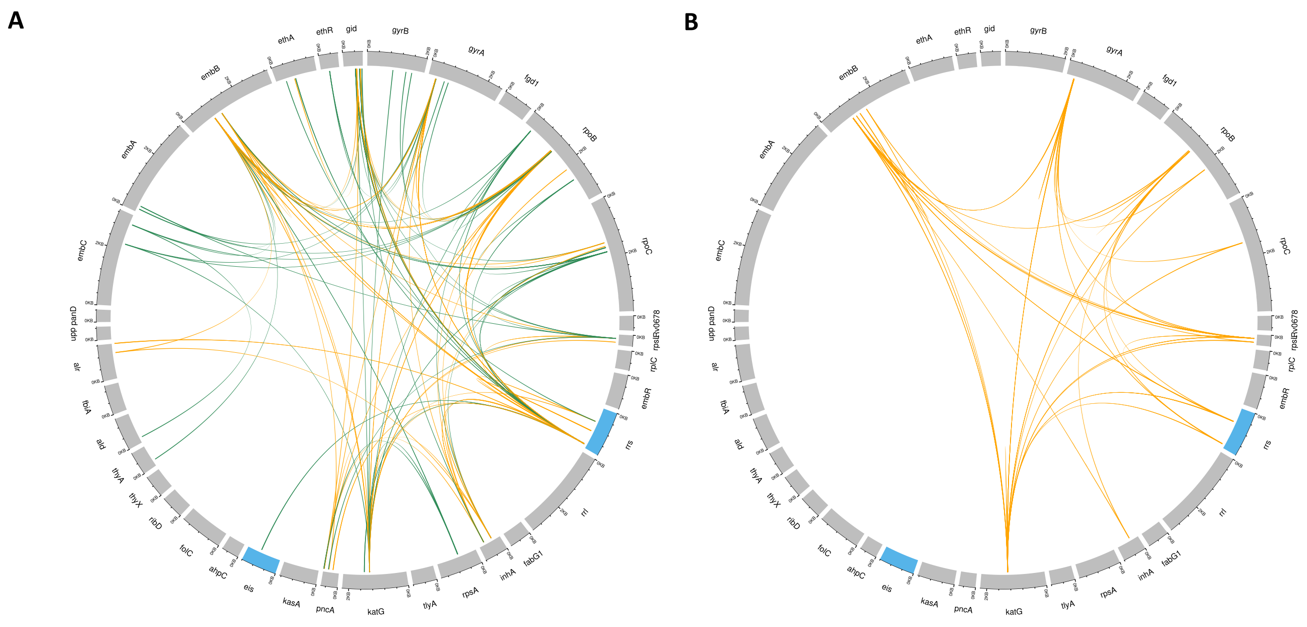

Supplementary Figure 18. Most frequent interactions (top 1%) observed across 1,000 trees in random forest model for kanamycin-resistance prediction. A) Most frequent variant-variant interactions in the unweighted model. B) Most frequent variant-variant interactions in the weighted model. Genes known to contain kanamycin-resistance mutations are highlighted in blue. Interactions are classified as a known drug-resistance interaction (blue), co-occurring interaction (orange) and lineage interaction (green).

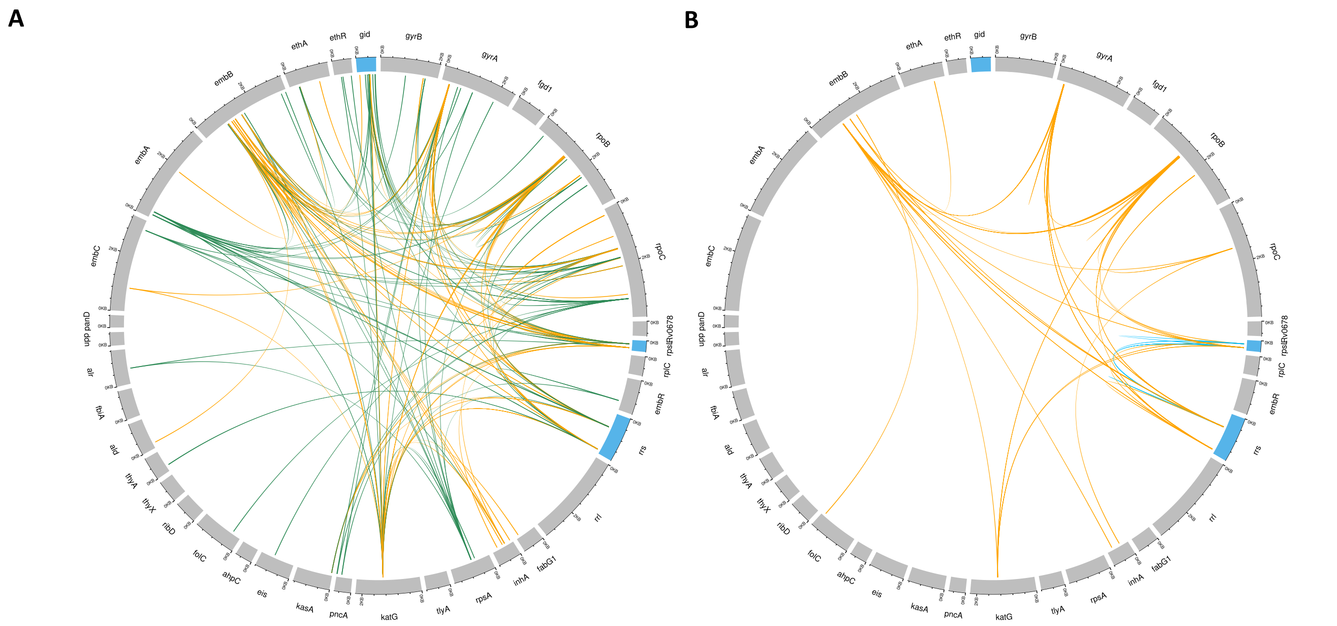

Supplementary Figure 19. Most frequent interactions (top 1%) observed across 1,000 trees in random forest model for streptomycin-resistance prediction. A) Most frequent variant-variant interactions in the unweighted model. B) Most frequent variant-variant interactions in the weighted model. Genes known to contain streptomycin-resistance mutations are highlighted in blue. Interactions are classified as a known drug-resistance interaction (blue), co-occurring interaction (orange) and lineage interaction (green).

A B

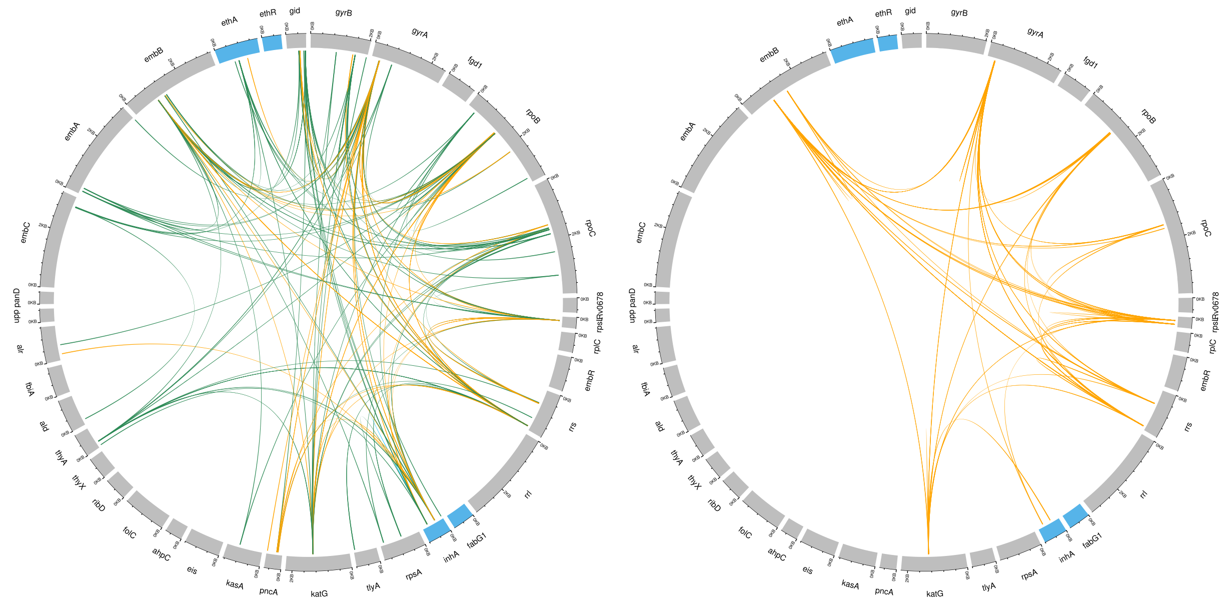

Supplementary Figure 20. Most frequent interactions (top 1%) observed across 1,000 trees in random forest model for ethionamide-resistance prediction. A) Most frequent variant-variant interactions in the unweighted model. B) Most frequent variant-variant interactions in the weighted model. Genes known to contain ethionamide-resistance mutations are highlighted in blue. Interactions are classified as a known drug-resistance interaction (blue), co-occurring interaction (orange) and lineage interaction (green).

A B

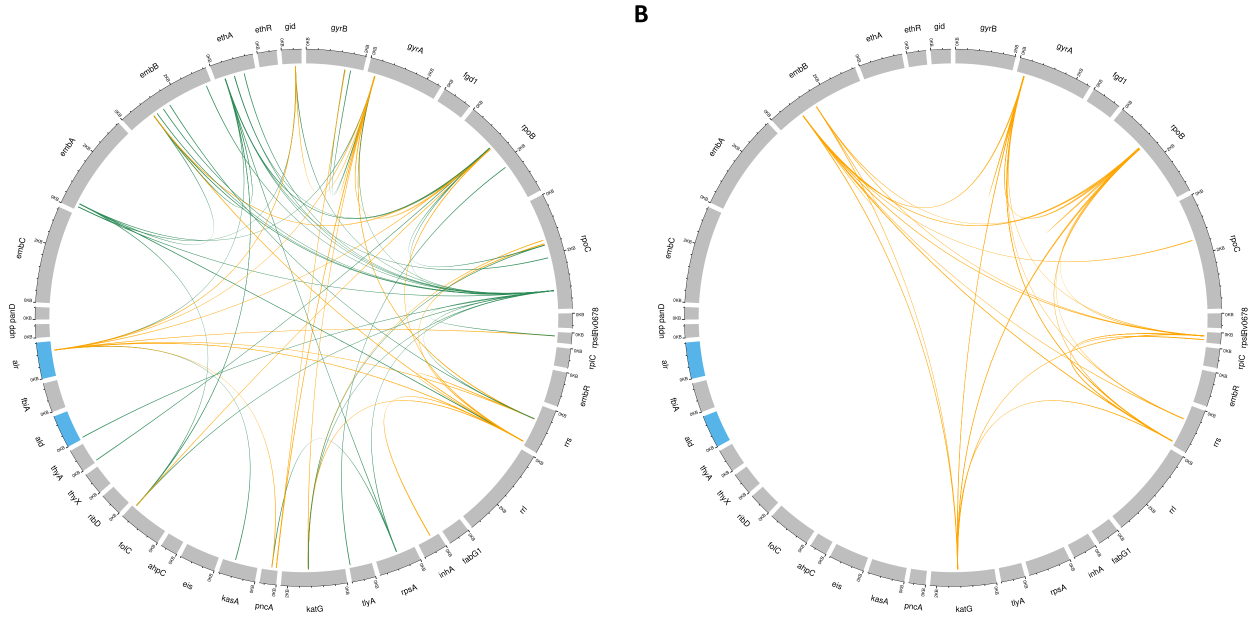

Supplementary Figure 21. Most frequent interactions (top 1%) observed across 1,000 trees in random forest model for cycloserine-resistance prediction. A) Most frequent variant-variant interactions in the unweighted model. B) Most frequent variant-variant interactions in the weighted model. Genes known to contain cycloserine-resistance mutations are highlighted in blue. Interactions are classified as a known drug-resistance interaction (blue), co-occurring interaction (orange) and lineage interaction (green).

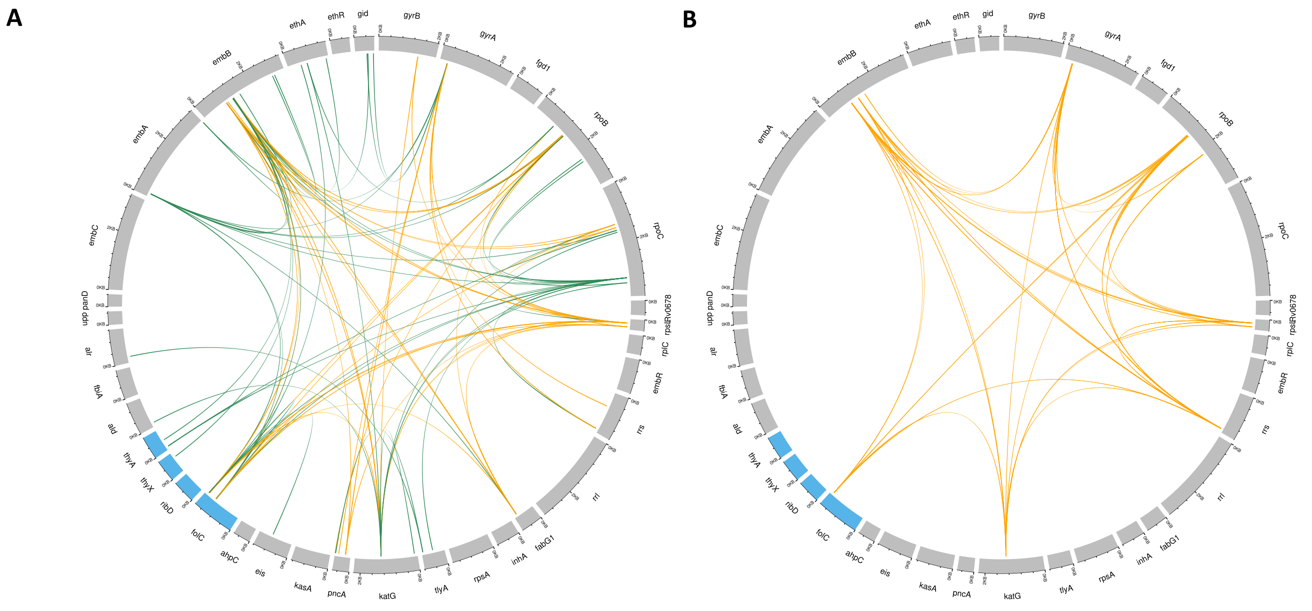

Supplementary Figure 22. Most frequent interactions (top 1%) observed across 1,000 trees in random forest model for para-aminosalicylic acid-resistance prediction. A) Most frequent variant-variant interactions in the unweighted model. B) Most frequent variant-variant interactions in the weighted model. Genes known to contain para-aminosalicylic acid-resistance mutations are highlighted in blue. Interactions are classified as a known drug-resistance interaction (blue), co-occurring interaction (orange) and lineage interaction (green).
